# Unveiling the Dual Mechanisms of *Solanum nigrum* L. Fractions Against Ovarian Cancer via AKT/NF-κB and Tumor Microenvironment Modulation: An In Silico and In Vitro Study

**DOI:** 10.3390/ijms27167076

**Published:** 2026-08-07

**Authors:** Eun-Ji Kang, Eun-Hye Kim, Hwi-Ho Lee, Yong-Deok Jeon, Jung-Hye Choi, Ji-Hye Ahn

**Affiliations:** 1 Department of Food science and Biotechnology, Woosuk University, Wanju 55338, Republic of Korea; kngj2642@naver.com; 2 Department of Korean Pharmacy, Woosuk University, Wanju 55338, Republic of Korea; kimune4307@gmail.com (E.-H.K.); hhlee4083@gmail.com (H.-H.L.); ydjeon1211jh@woosuk.ac.kr (Y.-D.J.); 3 Department of Biomedical and Pharmaceutical Sciences, Kyung Hee University, Seoul 02447, Republic of Korea; jchoi@khu.ac.kr

**Keywords:** *Solanum nigrum* L., network pharmacology, molecular docking, ovarian cancer, tumor microenvironment, tumor-associated macrophages, AKT/NF-κB pathway, apoptosis, invasion

## Abstract

Ovarian cancer treatment remains challenging due to aggressive metastasis and the complex tumor microenvironment (TME). *Solanum nigrum* L. (SN), a berry-bearing plant rich in bioactive phytochemicals, offers promising multi-target anticancer potential. This study elucidated the systemic molecular mechanisms of SN fractions against ovarian cancer by integrating network pharmacology, molecular docking, and in vitro models. In silico analyses and molecular docking identified apoptosis, metastasis-related pathways, and NF-κB (*RELA*) as core targets. For in vitro validation, butanol (SNBT) and methylene chloride (SNMC) fractions of SN were evaluated in SKOV3 cells. SNBT significantly induced caspase-3-dependent apoptosis, while SNMC effectively suppressed cancer cell migration and invasion without cytotoxicity. Furthermore, both fractions markedly downregulated phosphorylated AKT and NF-κB, experimentally confirming the suppression of the AKT/NF-κB signaling axis. Crucially, in a tumor-associated macrophage co-culture model, SNMC attenuated the pro-metastatic TME by reducing MMP2, MMP9, and VEGF secretion. Conclusively, SN exerts potent anticancer effects through distinct fractional activities, such as driving apoptosis and blocking metastasis, mediated via AKT/NF-κB inhibition and TME remodeling. These findings highlight SN-derived fractions as promising multi-target therapeutic candidates against ovarian cancer.

## 1. Introduction

Ovarian cancer (OVCA) represents a persistent and growing public health challenge [1]. Recent global estimates reported 324,398 newly diagnosed cases in 2022, and by 2050, the number of new cases is projected to increase by 55%, nearing half a million [2]. Despite advancements in surgical and chemotherapeutic treatments, survival rates have seen limited improvement. The high mortality of OVCA is primarily driven by asymptomatic progression, aggressive metastasis, and a highly complex tumor microenvironment (TME) that fosters drug resistance [3,4]. Within the TME, tumor-associated macrophages (TAMs) play a pivotal role in promoting tumor cell migration, invasion, and immune evasion [5]. Current first-line treatments, including platinum–taxane combinational therapies and recent targeted therapies (e.g., PARP inhibitors and immunotherapies), often fail to effectively remodel the TME and are frequently limited by severe systemic toxicities [6]. This highlights an urgent clinical need for novel, safer multi-target therapeutic options capable of concurrently inhibiting cancer cell survival and remodeling the pro-metastatic TME.

Medicinal plants and dietary phytochemicals have long served as a rich source of therapeutic agents, with their historical use offering valuable insights into clinical safety. Their inherent multi-component, multi-target mode of action stands in stark contrast to the conventional “one drug–one target” paradigm [7]. This conventional model is increasingly recognized as inadequate for addressing the complex, multi-factorial networks of diseases like OVCA [8]. Traditional multi-herb therapies provide a compelling example of this systems-level approach [9,10]. Although the chemical complexity of botanical preparations poses challenges for molecular validation, there is a burgeoning interest in employing network pharmacology and systems biology to decode the intricate pharmacological mechanisms underlying these natural products.

*Solanum nigrum* L. (SN) is a widely distributed berry-bearing plant that has been utilized for centuries in East Asian traditional medicine, particularly for detoxification and managing inflammatory conditions [11,12]. Modern pharmacological investigations have substantiated these properties, demonstrating the potent anticancer, anti-inflammatory, and antioxidant activities of its bioactive compounds [13,14]. Notably, crude extracts of SN have exhibited inhibitory effects on the proliferation of various cancers, including OVCA [15,16,17,18]. However, the majority of prior studies have focused primarily on isolated single molecules or general cell cytotoxicity using crude water extracts. Consequently, the specific roles of different SN fractions, their integrated multi-target signaling networks (e.g., the AKT/NF-κB axis), and their regulatory effects on the OVCA microenvironment and TAMs remain largely unexplored.

To bridge this significant knowledge gap, we employed an integrative strategy combining network pharmacology, molecular docking, and rigorous in vitro models. Through in silico predictions and molecular docking, we initially identified apoptosis, metastasis-related pathways, and NF-κB (RELA) as the core systemic targets of SN. To experimentally validate these network-level hypotheses, we sequentially fractionated the SN extract and investigated its fraction-specific pharmacological roles. Our in vitro validation demonstrated that these anticancer effects are molecularly driven by the suppression of the upstream AKT/NF-κB signaling axis. Specifically, the butanol fraction (SNBT) was evaluated for its apoptosis-inducing capacity via caspase activation, while the methylene chloride fraction (SNMC) was investigated for its anti-metastatic effects and TAM-mediated TME modulation. This work not only elucidates the comprehensive molecular mechanisms of SN but also highlights its potential as a promising multi-target candidate for disrupting the complex network of OVCA.

## 2. Results

### 2.1. Network Pharmacology Unveils Core Targets of SN Regulating Apoptosis and Migration in OVCA

**Figure 1:**
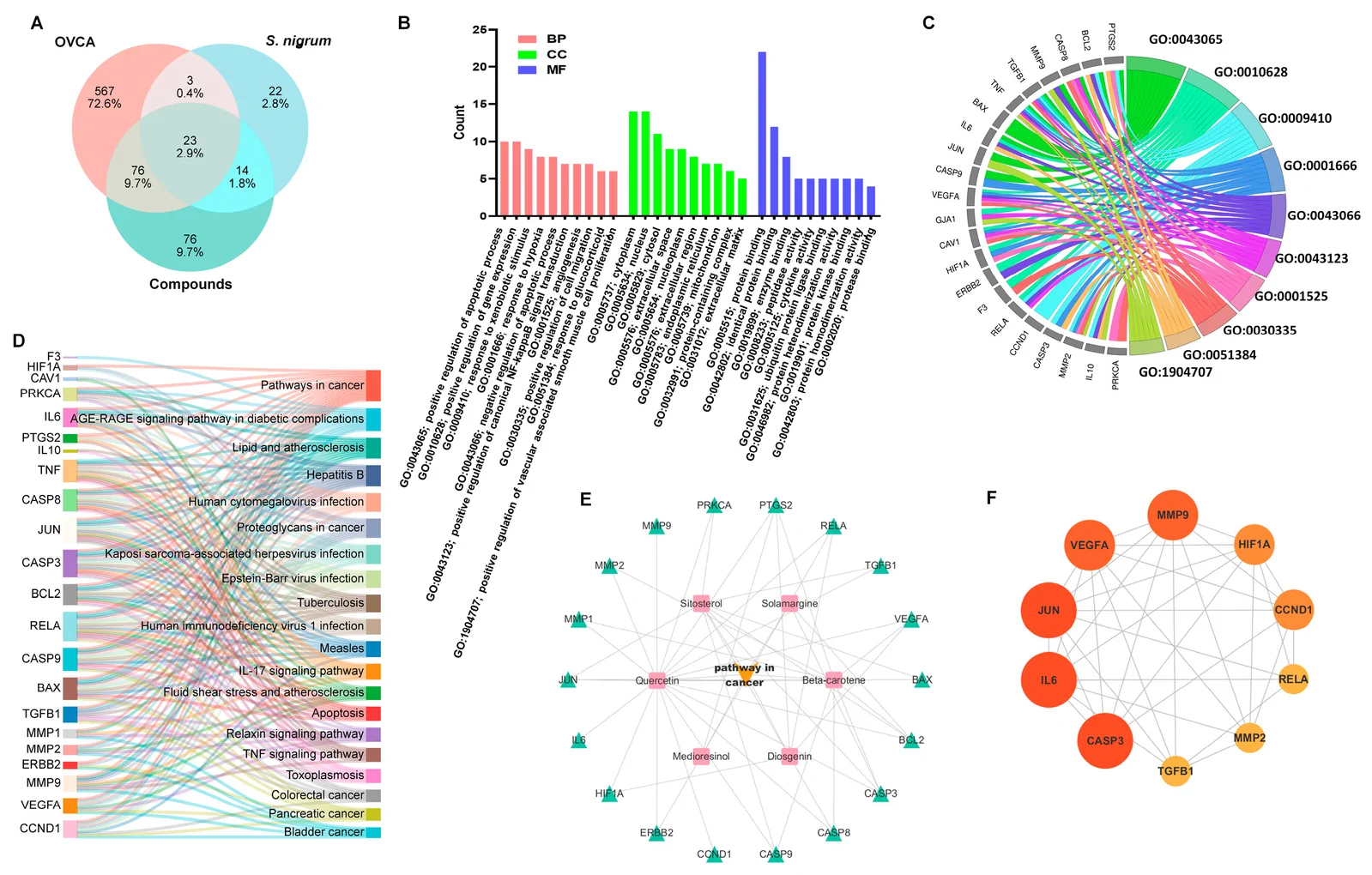
Network pharmacology analysis of SN against OVCA: (**A**) Venn diagram of 23 overlapping target genes among OVCA, the extract, and active compounds. (**B**) Top 10 enriched Gene Ontology (GO) terms for biological processes (BP, red), cellular components (CC, green), and molecular functions (MF, blue). (**C**) Chord diagram linking the 23 target genes to key GO terms. (**D**) Sankey diagram mapping the top 20 enriched KEGG pathways. (**E**) Network of 6 active compounds (pink), 18 target genes (light green), and the central “Pathways in cancer” pathway (orange). (**F**) Protein–protein interaction (PPI) network constructed via STRING (threshold > 0.700) and Cytoscape. Node size and color gradient (yellow to red) represent the degree centrality of the target genes.

Through the TCMSP database, nine active compounds in SN were identified. Seven compounds (diosgenin, medioresinol, solanocapsine, quercetin, cholesterol, beta-carotene, and sitosterol) met the selection criteria of OB ≥ 30% and DL ≥ 0.18, while two (solasonine and solamargine) were included based on substantial evidence of their biological activities (Table 1). Target prediction using Genecards, Malacards, and HERB databases revealed 669 OVCA-related genes (Table S1), 62 SN-associated genes (Table S2), and 189 compound-linked genes (Table S3). Intersection analysis identified 23 core targets shared across SN, its active compounds, and OVCA (Figure 1A).

To elucidate the biological roles of these targets, GO and KEGG enrichment analyses were conducted, which consistently highlighted pathways associated with cancer cell death and metastasis. In GO biological processes (BP), apoptosis and cell migration emerged as prominent terms (Figure 1B). Specifically, targets such as *BCL2, CASP8, MMP9, BAX*, and *CASP9* were heavily linked to the “positive regulation of apoptosis,” whereas *VEGFA, CAV1, CASP8, TGFB1, MMP2, PRKCA,* and *F3* were associated with the “positive regulation of cell migration” (Figure 1C and Table S4). Furthermore, KEGG analysis identified 107 significantly enriched pathways (*p* < 0.05), with “Pathways in Cancer” exhibiting the highest gene representation (18 targets, including *VEGFA, CASP8, BCL2, TGFB1, RELA, MMP9, CASP3, BAX,* and *MMP2*) (Figure 1D and Table S5). Interaction mapping confirmed that these 18 cancer-related genes are primarily regulated by six key SN compounds (β-carotene, diosgenin, medioresinol, quercetin, sitosterol, and solamargine) (Figure 1E and Table S6).

To prioritize the most critical therapeutic targets, Degree Centrality (DC) in the PPI network was analyzed, identifying 10 major hub genes: *CASP3, IL6, JUN, VEGFA, MMP9, HIF1A, CCND1, RELA, MMP2,* and *TGFB1* (Figure 1F and Table S7). Crucially, across all GO, KEGG, and PPI analyses, these central targets consistently clustered into three functional categories, namely, apoptosis executors (e.g., *CASP3/8/9, BAX, BCL2*), migration/metastasis mediators (e.g., *MMP2/9, VEGFA*), and upstream signaling regulators (e.g., *RELA/NF-κB*). Collectively, these systemic findings robustly indicate that SN exerts its anti-OVCA effects by modulating these specific apoptotic and migratory signaling nodes, providing a strong rationale for subsequent in vitro validations, specifically evaluating fraction-induced apoptosis and anti-migratory effects at non-toxic concentrations.

### 2.2. Fractionation Strategy Uncovers Divergent Cytotoxic Profiles and Enables Dual Validation of Apoptosis and Metastasis

Building upon our computationally predicted network, we sought to experimentally validate these multi-target hypotheses. Since crude plant extracts comprise complex mixtures of phytochemicals with varying solubilities, sequential fractionation based on solvent polarity is a standard strategy to isolate distinct bioactive pools and enhance specific biological activities [19,20]. Because our in silico analysis predicted both apoptotic and anti-metastatic mechanisms, we hypothesized that fractionating the crude extract would allow us to uncouple these distinct efficacies. Accordingly, the crude methanolic SN extract was sequentially partitioned into five sub-fractions: *n*-hexane, methylene chloride (SNMC), ethyl acetate, *n*-butanol (SNBT), and aqueous fractions. This strategic separation aimed to resolve the complex mixture into specific functional pools, enabling us to individually evaluate which fraction is responsible for inducing apoptosis and which regulates cell migration.

**Figure 2:**
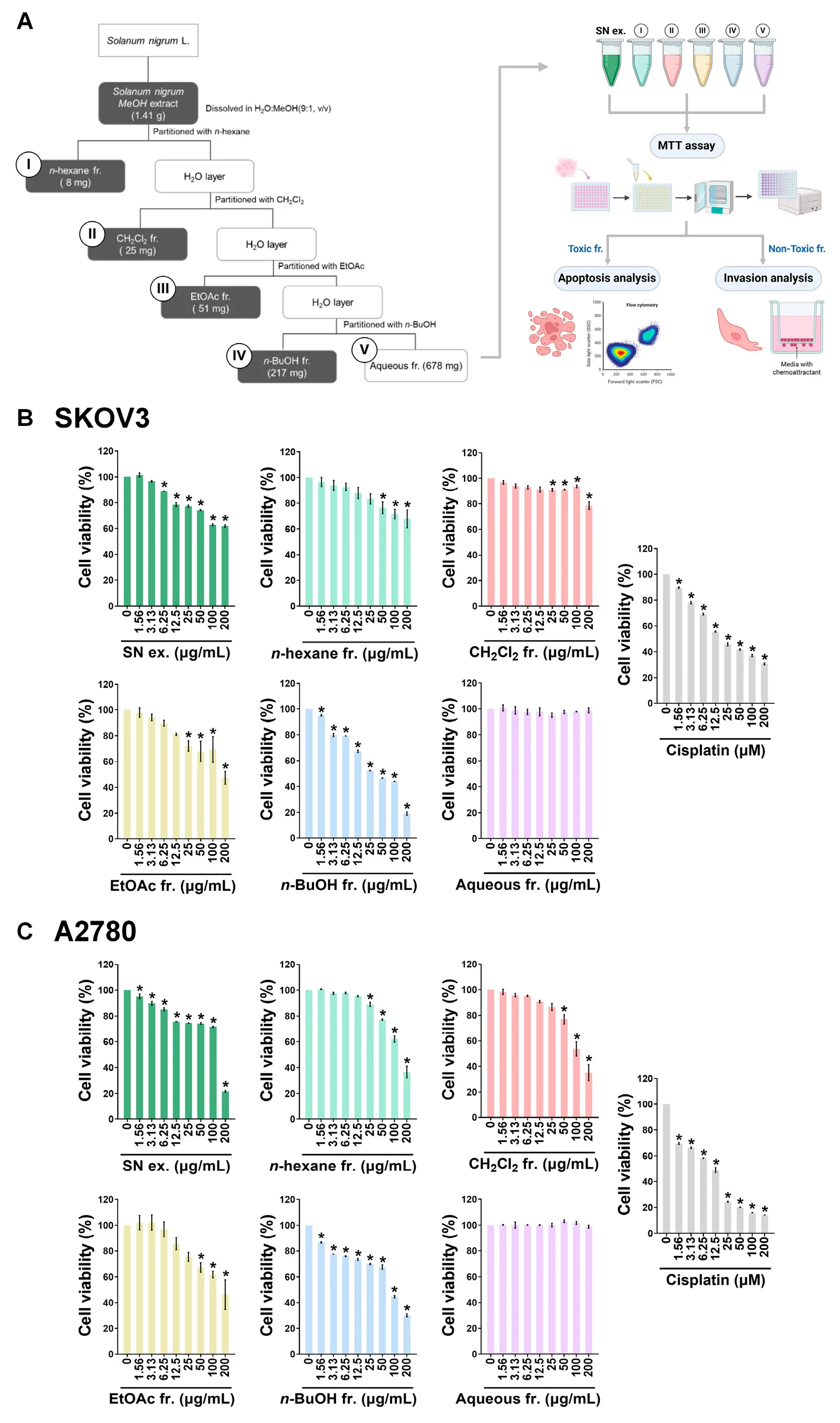
Solvent fractionation of SN and its cytotoxic effects on OVCA cells: (**A**) Schematic workflow of the sequential solvent fractionation process and the subsequent screening strategy. The fractions were categorized for either apoptosis or invasion analysis based on their cytotoxicity profiles. (**B**,**C**) Cell viability of SKOV3 (**B**) and A2780 (**C**) cells treated with varying concentrations (1.56–200 µg/mL) of the crude SN extract, its five distinct fractions (*n*-hexane, CH_2_Cl_2_, EtOAc, *n*-BuOH, and aqueous), and the positive control cisplatin (1.56–200 µM). Viability was determined using the MTT assay. Data are expressed as the mean ± standard deviation (SD) of three independent experiments. * *p* < 0.05 vs. the untreated control group.

To evaluate the biological profiles of these distinct pools, we conducted comprehensive cytotoxicity screenings across A2780 and SKOV3 ovarian cancer (OVCA) cell lines (Figure 2B,C). Interestingly, fractionation resulted in strikingly divergent pharmacological behaviors compared to the mildly cytotoxic total SN extract (IC50 > 200 µg/mL for SKOV3 and 111.50 ± 2.94 µg/mL for A2780; Table 2). Notably, the SNBT fraction emerged as the most potent, demonstrating significantly enhanced cytotoxicity, with IC50 values of 92.09 ± 4.12 µg/mL and 34.36 ± 0.06 µg/mL in A2780 and SKOV3 cells, respectively (Table 2). In contrast, the n-hexane and ethyl acetate fractions exhibited only moderate toxicity (IC50 ranging from 135.61 to > 200 µg/mL), while the SNMC and aqueous fractions maintained high cell viability, showing negligible cytotoxicity even at elevated concentrations (Table 2).

Based on this profound phenotypic divergence, we selected fractions with distinct pharmacological profiles for further targeted investigations. The highly cytotoxic SNBT fraction was chosen for subsequent apoptosis studies to evaluate its specific role in inducing programmed cell death. Conversely, the non-toxic nature of SNMC positioned it as the optimal candidate for investigating anti-migratory and anti-invasive properties independent of cellular toxicity. This selection directly aligned with our initial in silico predictions, establishing a targeted framework to validate the two major anticancer mechanisms of SN.

### 2.3. SNBT Dismantles the AKT and NF-κB Survival Axis to Execute Caspase-Dependent Apoptosis

**Figure 3:**
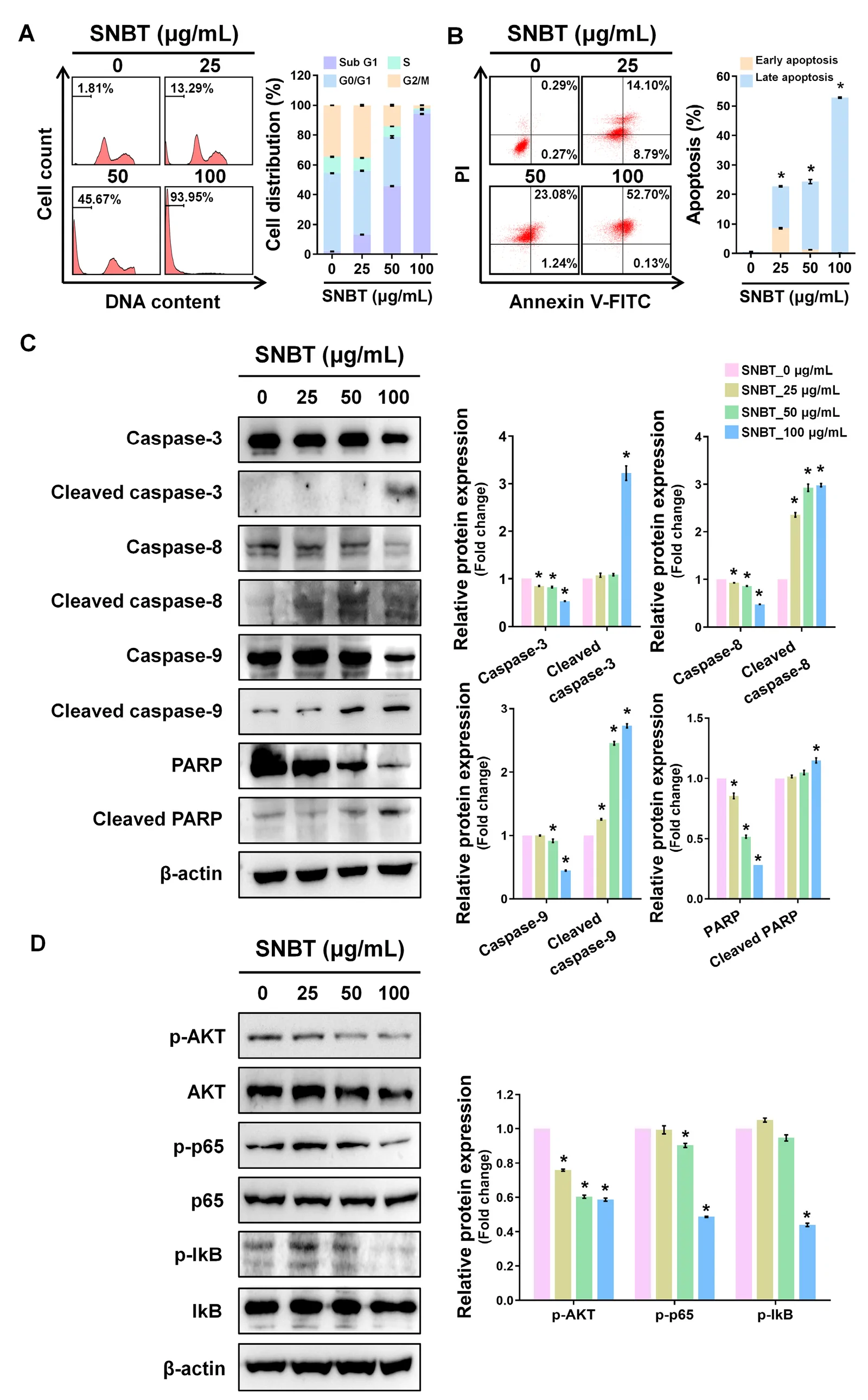
SNBT induces apoptosis and suppresses the AKT/NF-κB signaling axis in SKOV3 OVCA cells: (**A**,**B**) Representative flow cytometry plots and quantitative analysis of cell cycle distribution (**A**) and apoptosis (**B**) following treatment with varying concentrations of SNBT (0, 25, 50, and 100 μg/mL). (**C**) Western blot analysis and relative fold-change quantification of key apoptotic markers (caspase-3, caspase-8, caspase-9, PARP, and their cleaved forms). (**D**) Western blot analysis and quantification of key signaling molecules (AKT, p65, and IkB) and their phosphorylated active forms (p-AKT, p-p65, and p-IkB). β-Actin served as the loading control. Data are expressed as the mean ± SD of three independent experiments. * *p* < 0.05 vs. the untreated control.

Following the selection of SNBT based on its potent cytotoxic profile, we sought to elucidate the precise cellular mechanisms driving this activity and verify its alignment with the apoptotic networks predicted in silico. Cell cycle analysis revealed that SNBT treatment dramatically increased the sub-G1 population in a dose-dependent manner, a classical hallmark of DNA fragmentation (Figure 3A). Specifically, exposure to 50 µg/mL SNBT shifted 45.7% of the cell population into the sub-G1 phase, which further surged to 93.9% at 100 µg/mL. This profound phenotypic shift was independently corroborated via Annexin V and PI double staining (Figure 3B). The analysis demonstrated a substantial escalation in overall apoptotic rates, with 50 µg/mL SNBT inducing approximately 30% apoptosis and 100 µg/mL SNBT driving nearly 60% of the cells into apoptosis, predominantly localized in the late apoptotic phase.

Moving beyond these phenotypic observations, we interrogated the molecular executioners responsible for this programmed cell death. Western blot analysis confirmed that SNBT robustly activated both the intrinsic and extrinsic apoptotic cascades (Figure 3C). We observed a marked, dose-dependent accumulation of cleaved caspase-8 and cleaved caspase-9, which subsequently converged to activate the terminal executioner, caspase-3. The profound elevation of cleaved caspase-3 was accompanied by the complete cleavage of its downstream target, PARP. This coordinated, multi-level activation across the caspase cascade directly reflects the primary apoptosis executor functional category prioritized in our GO and KEGG predictions.

Crucially, to determine if this lethal cascade was driven by the suppression of the upstream regulatory nodes identified via network pharmacology, we evaluated the AKT and NF-κB signaling networks. As depicted in Figure 3D, SNBT treatment significantly and dose-dependently abrogated the phosphorylation of AKT, p65, and IκB. This simultaneous downregulation of the AKT and NF-κB survival axis provides compelling mechanistic evidence. It demonstrates that SNBT systematically dismantles the critical anti-apoptotic signaling hubs of OVCA, thereby perfectly validating the integrated computational model.

### 2.4. SNMC Abrogates OVCA Migration and Invasion by Disrupting EMT and the FAK-AKT-NF-κB Signaling Axis

**Figure 4:**
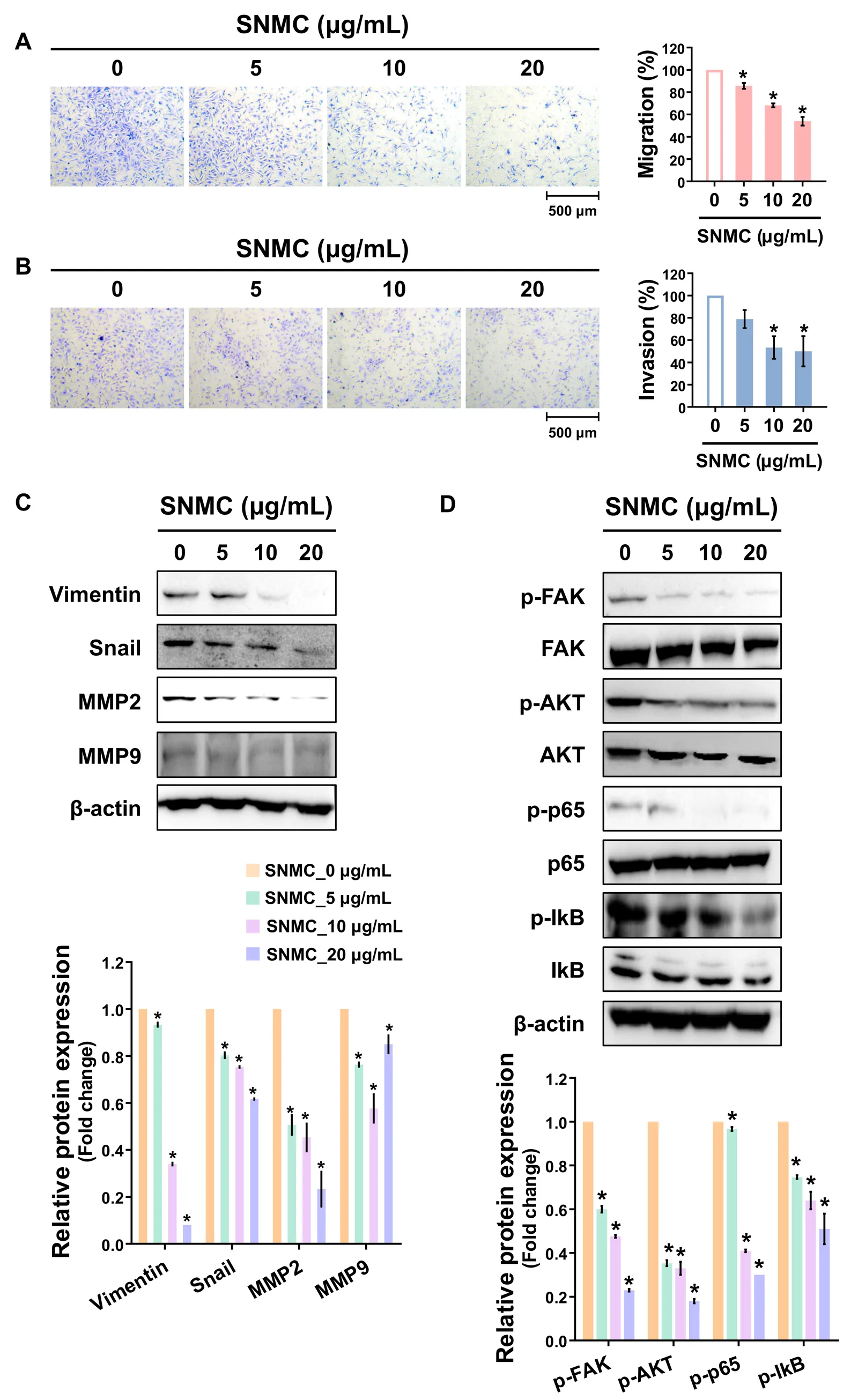
SNMC suppresses migration, invasion, and the FAK/AKT/NF-κB signaling axis in SKOV3 OVCA cells: (**A**,**B**) Representative images and quantitative analysis of cell migration (**A**) and invasion (**B**) following treatment with varying concentrations of SNMC (0, 5, 10, and 20 μg/mL). (**C**) Western blot analysis and relative fold-change quantification of EMT and metastasis markers (vimentin, Snail, MMP2, and MMP9). (**D**) Western blot analysis and quantification of key signaling molecules (FAK, AKT, p65, and IkB) and their phosphorylated active forms (p-FAK, p-AKT, p-p65, and p-IkB). β-Actin served as the loading control. Data are expressed as the mean ± SD of three independent experiments. * *p* < 0.05 vs. the untreated control.

In parallel with our apoptosis investigations, we utilized the non-toxic SNMC fraction to specifically interrogate the metastasis-related networks prioritized in our in silico analysis. As depicted in Figure 4A,B, SNMC treatment profoundly attenuated both the migratory and invasive capacities of SKOV3 cells. Crucially, these anti-metastatic effects were achieved at concentrations (5, 10, and 20 µg/mL; maintaining > 90% cell viability), ensuring that the observed phenotypic suppression was driven by genuine signaling interference rather than generalized cytotoxicity.

To decode the molecular effectors underlying this invasion blockade, we examined the expression of critical metastasis-associated proteins. Aligning perfectly with the network pharmacology predictions, SNMC orchestrated a marked, dose-dependent downregulation of key epithelial–mesenchymal transition drivers, specifically vimentin and Snail (Figure 4C). Concurrently, the fraction drastically suppressed the protein levels of MMP2 and MMP9, which were prioritized as core migratory targets in our in silico analysis and serve as the primary enzymatic effectors responsible for extracellular matrix degradation during OVCA cell invasion (Figure 4C).

Mechanistically, we traced these downstream alterations back to their upstream regulatory nodes. SNMC substantially inhibited the phosphorylation of focal adhesion kinase (FAK), a master regulator of cancer cell motility (Figure 4D). Furthermore, mirroring the profound signaling blockade observed with the SNBT fraction, SNMC effectively downregulated the activation of the AKT and NF-κB pathways, evidenced by decreased phosphorylation of AKT, p65, and IκB (Figure 4D). The striking convergence of both fractions on the AKT and NF-κB axis highlights this specific signaling cascade as the central, pleiotropic hub through which SN simultaneously halts metastasis via SNMC and drives apoptosis via SNBT, thereby robustly validating our systems-level predictions.

### 2.5. SNMC Remodels the TME by Neutralizing TAMs

**Figure 5:**
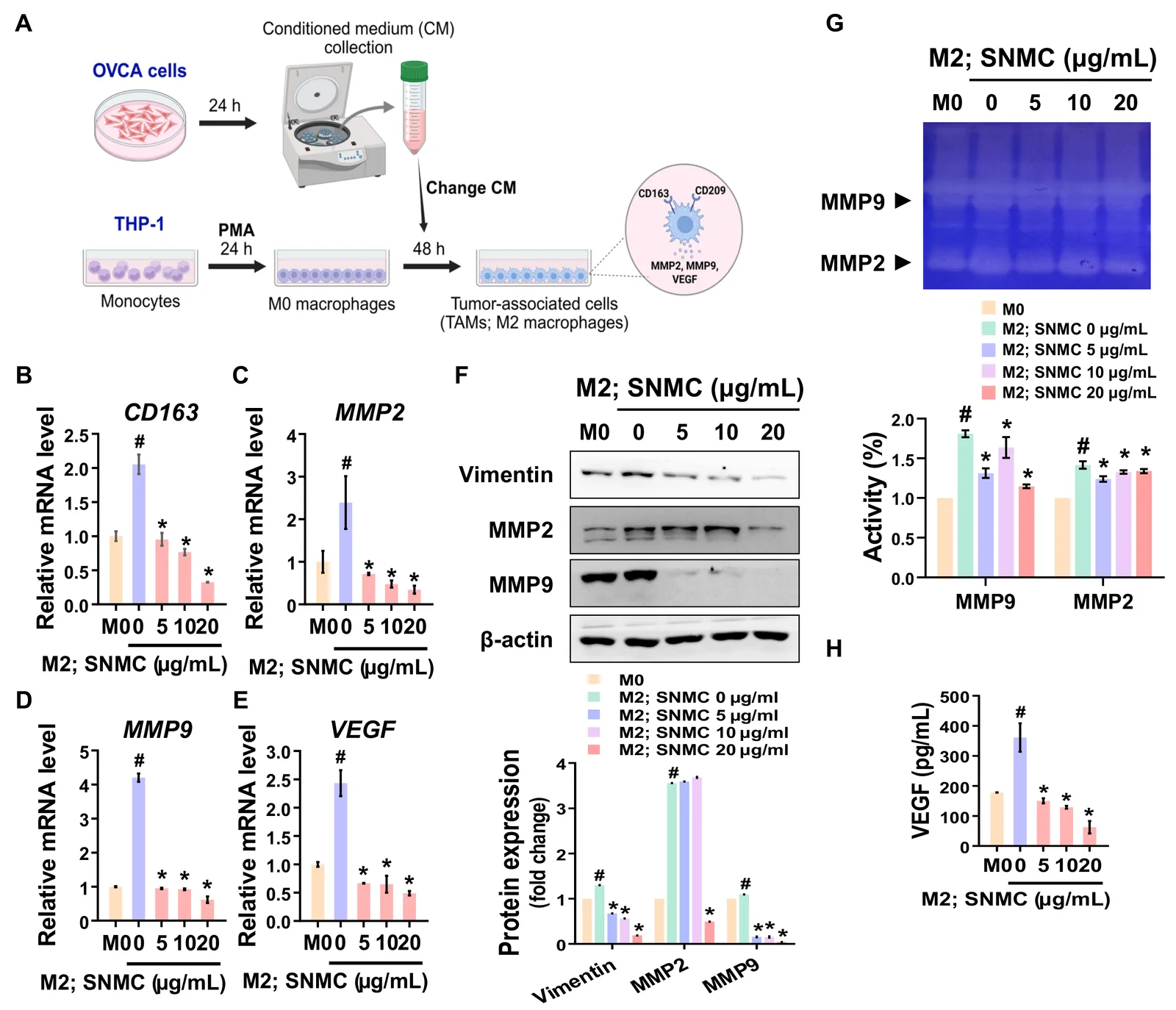
SNMC suppresses M2 macrophage polarization and the expression of pro-metastatic factors in THP-1 macrophages: (**A**) THP-1 cells were differentiated into TAM-like cells (M2) using SKOV3-conditioned medium, then treated for 48 h with SNMC (0, 5, 10, and 20 μg/mL). (**B**–**E**) Quantitative real-time PCR (qRT-PCR) analysis of relative mRNA levels for the M2 marker CD163 (**B**), MMP2 (**C**), MMP9 (**D**), and VEGF (**E**) in M0 or M2-polarized macrophages following treatment with varying concentrations of SNMC (0, 5, 10, and 20 μg/mL). (**F**) Western blot analysis and relative fold-change quantification of vimentin, MMP2, and MMP9. β-Actin served as the loading control. (**G**) Representative gel zymography images and quantitative analysis of MMP2 and MMP9 enzymatic activities. (**H**) ELISA quantification of secreted VEGF levels in the culture media. Data are expressed as the mean ± SD of three independent experiments. # *p* < 0.05 vs. the M0 control; * *p* < 0.05 vs. the untreated M2 control.

Beyond targeting isolated cancer cells, we investigated the therapeutic impact of SNMC on the tumor microenvironment. Within this niche, M2-polarized TAMs act as paracrine engines, fueling metastasis through the secretion of cytokines and matrix-degrading enzymes [21]. To evaluate this systemic efficacy, we employed an in vitro model of M2-polarized macrophages (Figure 5A). Strikingly, SNMC treatment significantly downregulated canonical M2-specific markers such as CD163 (Figure 5B), indicating a robust reversal of their tumor-promoting polarization state without inducing cellular toxicity.

Having established that SNMC disrupts macrophage polarization, we next evaluated its impact on the TAM-derived metastatic supply line. Quantitative PCR analysis revealed that SNMC profoundly diminished the mRNA expression of critical metastasis-promoting factors, specifically MMP2, MMP9, and VEGF (Figure 5C–E). This transcriptional repression was directly mirrored at the translational level. Western blot analysis demonstrated that SNMC markedly reduced the intracellular protein levels of MMP2 and MMP9 along with the mesenchymal marker vimentin within the TAMs (Figure 5F). These coordinated molecular alterations suggest that SNMC actively suppresses the inherent invasive machinery supported by the stromal compartment.

To definitively prove the functional neutralization of the TME, we assessed the extracellular activity of these secreted factors. Zymography assays confirmed that SNMC severely incapacitated the actual enzymatic activity of secreted MMP2 and MMP9 (Figure 5G), while ELISA demonstrated a drastic suppression of VEGF secretion into the extracellular space (Figure 5H). By effectively paralyzing these paracrine signaling networks, SNMC not only halts cancer cell motility directly but also restructures the surrounding microenvironment to become deeply hostile to metastasis. This dual compartmental efficacy highlights SNMC as a formidable systemic modulator capable of neutralizing the complex cellular crosstalk in OVCA.

### 2.6. Molecular Docking Validates Strong Physical Interactions of Solamargine with Core Functional and Signaling Hubs

**Figure 6:**
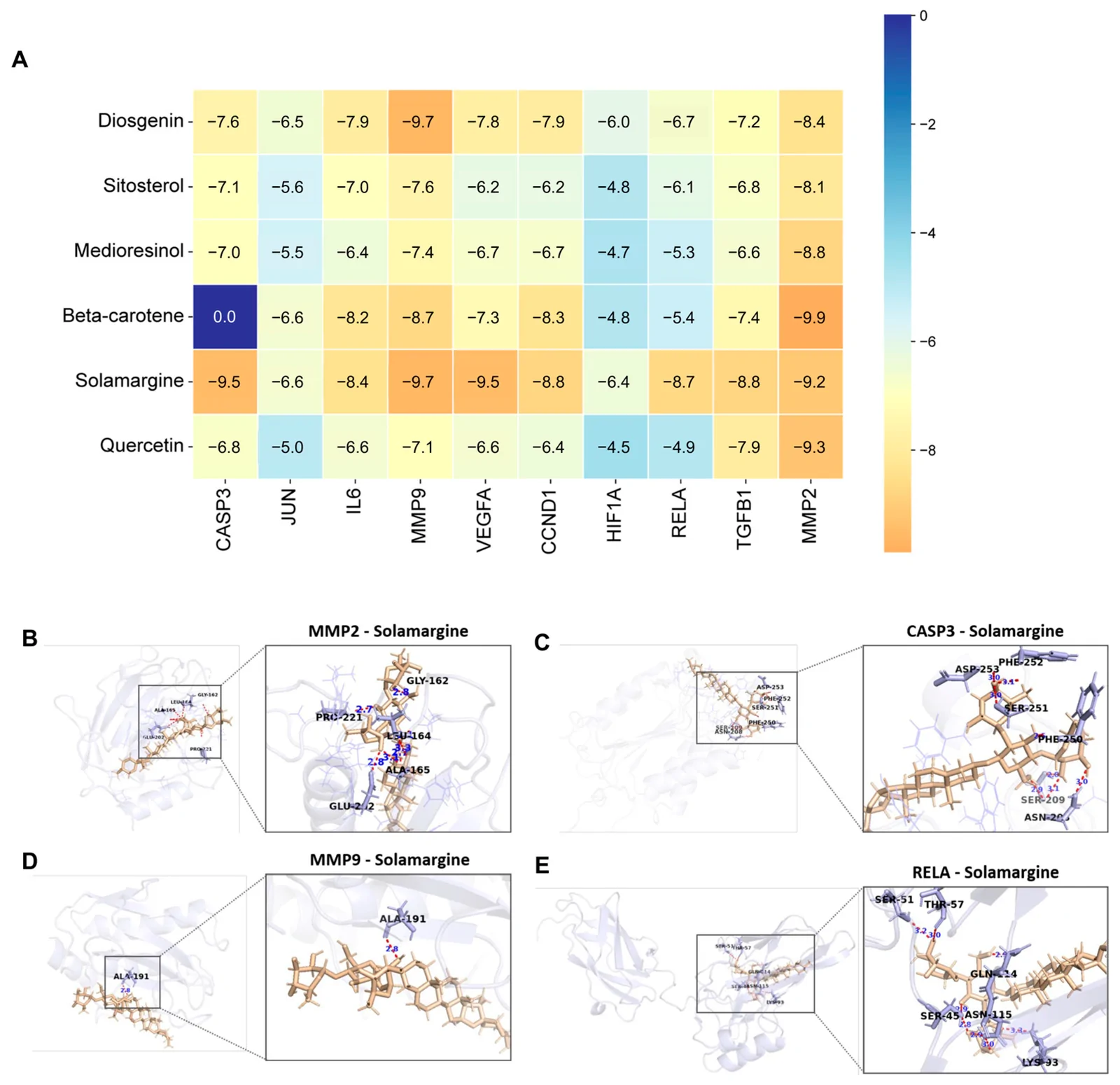
Molecular docking analysis of the key active compounds and core target proteins: (**A**) Heatmap of molecular docking binding energies (kcal/mol) between the six active compounds (diosgenin, sitosterol, medioresinol, beta-carotene, solamargine, and quercetin) and the top 10 core targets (CASP3, JUN, IL6, MMP9, VEGFA, CCND1, HIF1A, RELA, TGFB1, and MMP2). Lower values indicate stronger binding affinities. (**B**–**E**) The 3D visualizations of the optimal docking poses and molecular interactions of solamargine with selected key signaling and functional proteins, specifically MMP2 (**B**), CASP3 (**C**), MMP9 (**D**), and RELA (**E**). Magnified views detail the specific interacting amino acid residues and hydrogen bonds within the protein binding pockets.

To definitively establish the structural basis of the multi-compartmental efficacy observed in vitro, we ultimately sought to determine if our findings were driven by direct physical engagement between active SN compounds and the validated targets. Generally, binding energies below −5.0 kcal/mol denote stable interactions, while values below −7.0 kcal/mol signify exceptionally strong binding. As depicted in the comprehensive binding energy heatmap (Figure 6A), this computational analysis unveiled solamargine as the most prominent active compound, demonstrating profound binding avidity across all ten target proteins with energies ranging from −6.4 to −9.7 kcal/mol.

Specifically, the docking simulations corroborated the multi-level mechanism inferred from our functional assays. Solamargine orchestrated the deepest structural engagements with the key executioners of metastasis and apoptosis identified via network pharmacology. It exhibited the strongest binding affinity across all simulations, tightly docking into MMP9 (−9.7 kcal/mol). This was followed closely by a profound interaction with CASP3 (−9.5 kcal/mol), the central executioner of apoptosis, structurally reinforcing the contribution of solamargine to the potent cell killing observed with the SNBT fraction. Furthermore, it demonstrated exceptional affinity for MMP2 (−9.2 kcal/mol), providing the structural explanation for the dramatic TME remodeling. Crucially, addressing the common upstream regulatory axis, solamargine exhibited the strongest binding to RELA (−8.7 kcal/mol) among all six compounds. This definitive structural blockade perfectly mirrors the in vitro suppression of the AKT and NF-κB signaling networks observed across all experimental contexts.

Detailed 3D visualizations in Figure 6B–E confirmed these robust energy values with atomic-level precision, specifically illustrating the docking positions and bonding interactions between solamargine and the core functional protein hubs of MMP2, MMP9, CASP3, and RELA. These structural analyses revealed that solamargine tightly occupies the active pockets of these critical target proteins, stabilized by a dense and intricate network of extensive hydrogen bonds and deep hydrophobic contacts. Collectively, these docking results provide a robust mechanistic capstone for our study. They offer definitive structural evidence that the potent multi-target anticancer efficacy of SN is fundamentally driven by the direct and simultaneous physical engagement of its primary active molecule, solamargine, with the critical upstream signaling regulators and downstream functional effectors of OVCA.

## 3. Discussion

The treatment of OVCA remains a formidable clinical challenge, largely due to its complex molecular heterogeneity, frequent recurrence, and the rapid development of chemoresistance [22,23,24]. Conventional single-target therapeutic strategies often fall short in addressing the systemic nature of OVCA progression [8]. Consequently, there is a growing paradigm shift towards exploring multi-target botanical therapeutics that can simultaneously modulate various signaling networks. In this context, SN has emerged as a promising candidate. However, the precise systemic mechanisms underlying its anti-OVCA efficacy have remained largely elusive. In the present study, we integrated network pharmacology, molecular docking, and functional in vitro validations to systematically decode the pleiotropic anticancer properties of SN.

A primary strength of the present study is the experimental separation of the dual therapeutic activities of SN through solvent-guided fractionation. Profiling the distribution of major active constituents across sub-fractions is a widely accepted methodology to elucidate the chemical basis of biological activities in botanical research [25]. Accordingly, we quantified the concentration of solasonine, a well-known bioactive alkaloid of SN, within the crude extract and its five fractions (Figure S1 and Table S8). The observed pharmacological phenotypes did not demonstrate a linear correlation with solasonine abundance. Although the *n*-hexane fraction contained the highest solasonine concentration (0.740 mg/g^2^), it exhibited substantially lower cytotoxicity than the SNBT fraction, which contained only 0.216 mg/g^2^ of solasonine. In addition, the SNMC fraction effectively suppressed cell migration and invasion despite possessing a marginal solasonine content (0.045 mg/g^2^) and lacking cellular toxicity. This observation suggests that the overall biological efficacy of SN may not be solely attributed to a single dominant molecule. Instead, these results indicate alignment with the multi-component and multi-target paradigm of botanical medicines [26]. The potent activities of the SNBT and SNMC fractions likely result from synergistic or additive interactions among various phytochemicals co-eluting within those specific polarity ranges.

Our comparative analysis with cisplatin highlights a distinct clinical advantage of SN concerning cellular safety and target selectivity. Cisplatin remains a highly potent chemotherapeutic agent for ovarian cancer, but severe off-target toxicity frequently limits its clinical application [27]. The in vitro data of the present study reflected this limitation, as cisplatin induced non-selective cytotoxicity in both OVCA cells and normal ovarian epithelial cells. By contrast, the SN extract and its sub-fractions exhibited robust tumor-specific activity while preserving normal cellular viability (Figure S2). It is noteworthy that SNBT yielded potent cytotoxicity approaching clinically relevant levels despite being a complex phytochemical mixture, while the non-toxic SNMC fraction independently provided significant anti-metastatic benefits. Taken together, these findings suggest that SN possesses considerable potential as a safe and dual-targeted therapeutic candidate capable of simultaneously promoting apoptosis and impeding metastasis without the severe adverse effects typically associated with conventional chemotherapy.

To elucidate the molecular mechanisms underlying these distinct biological activities, we investigated key upstream signaling cascades. Both SNBT and SNMC effectively inhibited the AKT and NF-κB signaling pathways. This indicates that they dismantle the same upstream regulatory networks required for cancer cell survival and progression. This shared systemic blockade is further supported by our molecular docking results, which identified solamargine as the primary active modulator. Chemically, solamargine is a major steroidal glycoalkaloid in SN that preferentially partitions into polar solvents [28], enriching the highly cytotoxic SNBT fraction. Previous studies have firmly established that solamargine exerts potent pro-apoptotic effects in various cancers by modulating the PI3K/AKT and NF-κB signaling pathways [29,30]. Furthermore, our structural docking revealed that solamargine exhibits exceptionally strong binding affinities to key metastasis mediators, MMP2 and MMP9. This structural avidity perfectly aligns with recent findings that solamargine significantly inhibits migration and invasion of human hepatocellular carcinoma cells by directly downregulating MMP2 and MMP9 expression and enzymatic activity [31]. Our results provide a robust structural rationale for how solamargine exerts these dual effects.

Beyond direct cytotoxicity against isolated cancer cells, a major hallmark of our study is the demonstration of SN’s efficacy within the TME. In ovarian cancer, the TME is notoriously immunosuppressive and pro-metastatic, largely driven by the infiltration and M2 polarization of TAMs [32,33]. These TAMs establish a bidirectional paracrine signaling loop with cancer cells, continuously secreting extracellular matrix-degrading enzymes (MMP2 and MMP9) and angiogenic factors (VEGF) to facilitate tumor dissemination [34,35]. Addressing this complex stromal crosstalk is increasingly recognized as a prerequisite for effective OVCA therapy [36]. Our findings reveal that the non-toxic SNMC fraction effectively disrupts this pro-tumoral niche. Importantly, SNMC profoundly downregulated M2-specific markers (e.g., CD163) and suppressed the expression, protein translation, and extracellular secretion of MMP2, MMP9, and VEGF in TAMs, notably without inducing macrophage cell death. Modulating the TME by reprogramming rather than depleting macrophages represents a highly desirable therapeutic strategy. It neutralizes the pro-metastatic supply line and restores tissue homeostasis while avoiding the inflammatory consequences often associated with massive myeloid cell death. Furthermore, the strong physical binding of solamargine to MMP2 and MMP9 provides a clear mechanistic basis for the diminished enzymatic activities observed in our TME model. This dual action targeting both cancer cells and their supporting microenvironment aligns perfectly with the principles of integrative oncology.

Despite the significant insights provided by this integrated approach, it is important to acknowledge certain limitations. Our experimental validations were conducted exclusively using in vitro cell models, which cannot fully replicate the physiological complexity and immune dynamics of an in vivo TME. Future in vivo studies are essential to comprehensively evaluate these TME-remodeling effects and confirm their therapeutic efficacy. Additionally, although molecular docking identified solamargine as the primary active compound, our functional assays utilized complex phytochemical fractions. The observed biological effects could therefore result from synergistic interactions among multiple constituents. Subsequent research should focus on the isolation and empirical testing of solamargine to definitively confirm its individual and combined therapeutic roles. Nevertheless, the current findings provide compelling evidence for the distinct clinical advantages of SN. By demonstrating potent anticancer efficacy that approaches conventional chemotherapeutics like cisplatin while strictly preserving the viability of normal epithelial cells, SN effectively addresses the critical challenge of off-target toxicity. Ultimately, this foundational research supports the continued exploration of SN as a safe and highly effective multi-target therapeutic candidate capable of simultaneously driving apoptosis and suppressing metastasis to overcome the formidable challenges of current ovarian cancer treatments.

## 4. Materials and Methods

### 4.1. Reagents and Cell Lines

Solasonine (purity > 98%) was obtained from Chemfaces (Wuhan, China). Human ovarian cancer cell lines (SKOV3 and A2780) and the human monocyte cell line THP-1 were purchased from the American Type Culture Collection (ATCC, Manassas, VA, USA). Human immortalized ovarian surface epithelial cells (IOSE80PC) were kindly provided by Dr. N. Auersperg (University of British Columbia, Vancouver, BC, Canada) and Dr. A. Godwin (Fox Chase Cancer Center, Philadelphia, PA, USA). Reagents including 3-(4,5-dimethylthiazol-2-yl)-2,5-diphenyltetrazolium bromide (MTT) and propidium iodide (PI) were sourced from Sigma-Aldrich (St. Louis, MO, USA). Antibodies targeting FAK, Snail, p65, IκB, and β-actin were acquired from Santa Cruz Biotechnology (Santa Cruz, Dallas, TX, USA). Antibodies for phospho-FAK, phospho-IκB, MMP2, MMP9, caspase-3, caspase-8, caspase-9, and PARP were supplied by Cell Signaling Technology (Danvers, MA, USA). Antibodies against vimentin, phospho-AKT, AKT, and phospho-p65 were purchased from CUSABIO (Houston, TX, USA).

### 4.2. Preparation and Solvent Fractionation of SN Extract

Aerial parts of SN were procured from GwangMyngDang Oriental Medicine Pharmaceutical Co. (Ulsan, Republic of Korea) and authenticated with a voucher specimen (WSKP-2025-SN07) deposited at Woosuk University. For the extraction, 40 g of dried powdered aerial parts were soaked in 400 mL of 80% methanol for 24 h at room temperature, followed by reflux extraction at 80 °C for 3 h. The combined supernatants were filtered, evaporated under reduced pressure at 65 °C, and lyophilized for 72 h to yield 7.6 g of dried methanolic extract (19% yield). For solvent fractionation, 1 g of the lyophilized extract was dissolved in a distilled water and methanol mixture (9:1, *v*/*v*) and subjected to successive liquid–liquid partitioning using solvents of increasing polarity. This yielded n-hexane (8 mg), methylene chloride (SNMC, 25 mg), ethyl acetate (51 mg), n-butanol (SNBT, 217 mg), and aqueous (678 mg) fractions. Fraction identities were validated via UHPLC-QE-MS using solasonine as a reference (Figure S1).

### 4.3. Network Pharmacology and In Silico Target Prediction

Active compounds of SN were screened using the Traditional Chinese Medicine Systems Pharmacology (TCMSP) database based on oral bioavailability (OB ≥ 30%) and drug-likeness (DL ≥ 0.18) thresholds. Solamargine and solasonine were additionally included due to their established biological significance. Ovarian cancer-related targets were retrieved from the GeneCards and MalaCards databases, while the targets of the selected SN compounds were acquired from the HERB database. Overlapping genes were identified and utilized to construct a protein–protein interaction (PPI) network via the STRING database (confidence score > 0.700). Network visualization and topological analyses (Degree, Betweenness, and Closeness Centralities) were performed using Cytoscape (v3.10.2) with the CentiScaPe plugin. Subsequently, GO biological process and KEGG pathway enrichment analyses were conducted using the DAVID database (*p* < 0.05), and data were visualized using SRplot.

### 4.4. Molecular Docking Simulation

To validate physical interactions, 3D structures of the active SN compounds were generated from SMILES strings obtained via PubChem. The 3D crystal structures of the core hub targets identified in the PPI analysis were retrieved from the RCSB Protein Data Bank (PDB). Molecular docking simulations to calculate binding affinities were executed using AutoDock Vina (version 1.2.1). The stability and specific bonding interactions of the resulting complexes were visualized in 3D using PyMOL (version 1.8.5) and mapped in 2D using LigPlot+ (version 2.2).

### 4.5. Cell Culture and In Vitro TME Model

SKOV3, A2780, and IOSE800PC cells were cultured in RPMI 1640 medium (Welgene, Gyeongsan-si, Republic of Korea) supplemented with 5% fetal bovine serum (FBS) and antibiotics at 37 °C with 5% CO_2_. THP-1 monocytes were cultured in RPMI 1640 containing 10% FBS and 0.05 mM 2-mercaptoethanol. To establish the in vitro TME model, THP-1 cells were first differentiated into resting macrophages using 100 nM phorbol myristate acetate (PMA) for 48 h. These macrophages were subsequently polarized into TAMs by incubation with conditioned medium derived from SKOV3 cells for an additional 48 h.

### 4.6. Cell Viability Assay

To investigate the cytotoxic effects of the SN extract and its various solvent fractions on OVCA cells, an MTT assay was conducted. SKOV3 (0.7 × 10^5^ cells/mL), A2780 (1.0 × 10^5^ cells/mL), and IOSE80PC (0.7 × 10^5^ cells/mL) cells were seeded into 96-well plates and incubated for 24 h. The cells were then treated with the SN extract and fractions for 48 h. Following incubation, 15 μL of MTT solution (5 mg/mL) was added to each well, and the plates were incubated for an additional 4 h. The supernatant was removed, and the formazan crystals were dissolved in dimethyl sulfoxide. Absorbance was measured at 560 nm to quantify cell viability. For the OVCA cell lines (SKOV3 and A2780), the results represent three independent biological replicates (n = 3), whereas for the normal epithelial cell line (IOSE80PC), the results are derived from two biological replicates (n = 2), each comprising three technical replicates.

### 4.7. Cell Cycle Analysis

To evaluate cell cycle progression, SKOV3 cells were washed twice with cold PBS and then fixed and permeabilized in 75% ethanol at 4 °C overnight. Following fixation, the cells were washed twice with cold PBS, resuspended in PI solution (50 μg/mL) containing RNase A (250 μg/mL), and incubated for 20 min at room temperature in the dark. The cell cycle distribution was analyzed by detecting 10,000 cells per sample using a Guava easyCyte Flow Cytometer (Cytek Biosciences, Fremont, CA, USA). The cell cycle distribution was analyzed across three independent biological replicates (n = 3).

### 4.8. Apoptosis Analysis

Apoptosis was assessed using an Annexin V Apoptosis Detection Kit (eBioscience, San Diego, CA, USA). SKOV3 cells were rinsed with cold PBS and then double-stained with Annexin V and PI following the manufacturer’s protocol. After staining, the rate of apoptosis was quantified by analyzing 10,000 cells per sample using a Guava easyCyte Flow Cytometer. Apoptosis quantification was independently repeated in three biological replicates (n = 3).

### 4.9. Migration and Invasion Assays

A 24-well Transwell insert (8 μm pore size) with a PVPF filter was used to evaluate the inhibitory effect of SNMC on the migratory and invasive abilities of SKOV3 cells. For the migration assay, cells in RPMI 1640 medium supplemented with 1% FBS were seeded into the upper chambers. The cells were treated with SNMC (5, 10, and 20 μg/mL) and incubated for 24 h. For the invasion assay, cells were seeded into Matrigel-coated upper chambers and treated with SNMC for 48 h. Following incubation, the cells that migrated or invaded through the membrane were fixed with methanol for 10 min and stained with 0.2% crystal violet for 20 min. Non-migratory or non-invading cells on the upper surface were removed using a cotton swab. The migrated or invaded cells were quantified by counting five randomly selected fields per filter under an inverted microscope. Both assays were performed in three independent biological replicates (n = 3).

### 4.10. Western Blot Analysis

Prior to protein extraction, OVCA cells (SKOV3) were treated with indicated concentrations of SNBT (0, 25, 50, and 100 μg/mL) or SNMC (0, 5, 10, and 20 μg/mL) for 24 or 48 h, whereas M2-polarized TAMs were treated with SNMC (0, 5, 10, and 20 μg/mL) for 48 h. The cells were lysed using a protein lysis buffer (Intron Biotechnology, Seoul, Republic of Korea) containing a protease inhibitor cocktail, phosphatase inhibitor cocktail, and phenylmethylsulfonyl fluoride. The lysate was mixed with SDS sample buffer and boiled at 95 °C for 5 min before being separated by SDS-PAGE. Following electrophoresis, proteins were transferred to nitrocellulose membranes. The membranes were blocked with 5% skimmed milk at room temperature for 30 min, washed with TBS-T, and incubated overnight at 4 °C with primary antibodies (1:1000). The membranes were then washed three times with TBS-T and incubated with secondary antibodies (1:500 or 1:1000) at room temperature for 2 h. Immunopositive bands were visualized using enhanced chemiluminescence and detected with a LuminoGraph III Lite imager (Atto, Tokyo, Japan). All Western blot analyses were performed using lysates from three independent biological experiments (n = 3).

### 4.11. Quantitative Real-Time RT PCR

M2-polarized TAMs were treated with varying concentrations of SNMC (0, 5, 10, and 20 μg/mL) for 24 h prior to RNA extraction. Total RNA was extracted from cells using Trizol Reagent (Invitrogen, Carlsbad, CA, USA) following the manufacturer’s protocol. Subsequently, 500 ng of total RNA was reverse-transcribed into cDNA using a first-strand synthesis kit (Enzynomics, Daejeon, Republic of Korea). Quantitative real-time RT-PCR was conducted using TB Green Premix EX Taq (TaKaRa, Kyoto, Japan). The PCR cycle conditions were set to 95 °C for 10 s (denaturation), 60 °C for 10 s (annealing), and 72 °C for 10 s (extension). The cycle threshold values for each target gene were normalized to the expression of APP, which served as the internal reference. The specific primer sequences used were as follows: CD163 (sense: 5′-AGCAGGGATGTTGGAGTAGT-3′, anti-sense: 5′-TAAGCTGCTGGCAAAGAACA-3′), MMP2 (sense: 5′-TGATCTTGACCAGAATACCATCGA-3′, anti-sense: 5′-GGCTTGCGAGGGAAGAAGTT-3′), MMP9 (sense: 5′-TCTACACCCAGGACGGCAAT-3′, anti-sense: 5′-GAAGCCGAAGAGCTTGTCCC-3′), VEGF (sense: 5′-AGGTAGCAAGAGCTCCAGAG-3′, anti-sense: 5′-CCCAAAAAGCAGGTCACTCAC-3′), and APP (sense: 5′-GTGAAGATGGATGCAGAATTCCG-3′, anti-sense: 5′-AAAGAACTTGTAGGTTGGATTTTCG-3′). All qRT-PCR reactions were performed in three independent biological replicates (n = 3), with each reaction run in technical duplicate.

### 4.12. Functional Assays for Secreted Factors

The functional activity of TME-associated factors secreted by TAMs was evaluated using conditioned media following SNMC treatment. MMP2 and MMP9 enzymatic activities were measured via gelatin zymography. Concentrated media samples were separated on 8% SDS-PAGE gels copolymerized with 1% gelatin. The gels were renatured in 2.5% Triton X-100, incubated in collagenase buffer at 37 °C for 24 h, and stained with Coomassie blue to reveal zones of clearance indicating enzymatic activity. Additionally, the secretion levels of VEGF in the TAM-conditioned media were quantified using a Human VEGF ELISA Kit (Koma Biotech, Seoul, Republic of Korea) according to the manufacturer’s protocol. Both gelatin zymography and ELISA were conducted using conditioned media obtained from three independent biological replicates (n = 3).

### 4.13. Statistical Analysis

All experimental data are presented as the mean ± standard deviation (SD) derived from at least three independent experiments. Statistical significance was evaluated using one-way or two-way analysis of variance (ANOVA), depending on the experimental design, followed by Tukey’s post hoc test for multiple group comparisons. Statistical analyses and graphical representations were performed using GraphPad Prism 8 software. A *p*-value of < 0.05 was considered statistically significant.

## 5. Conclusions

This study successfully identifies the multi-target anticancer mechanisms of SN against ovarian cancer through the integration of network pharmacology and functional experimental validation. Through solvent fractionation, we evaluated the dual therapeutic effects of the extract. The SNBT fraction induced caspase-dependent apoptosis, while the non-toxic SNMC fraction suppressed cancer cell metastasis and inhibited the pro-tumoral activity of M2 macrophages. Both fractions exerted their effects by suppressing the AKT and NF-κB signaling pathways. Furthermore, molecular docking analysis indicated that solamargine is a key active compound that stably binds to core target proteins, including CASP3, MMP2, MMP9, and RELA. These findings demonstrate that the extract represents a promising multi-target therapeutic strategy that directly induces cancer cell death and restructures the supporting tumor microenvironment. Notably, unlike cisplatin, the extract and its active fractions exhibited no cytotoxicity toward normal ovarian epithelial cells, demonstrating a distinct clinical advantage in safety. Ultimately, these results support the continued exploration of SN as a safe and effective candidate to overcome current ovarian cancer treatment limitations.

## Figures and Tables

**Table 1:** Nine active compounds from SN satisfying TCMSP criteria for oral bioavailability (OB ≥ 30%) and drug-likeness (DL ≥ 0.18).

Mol ID	Molecule Name	MW	OB (%)	DL
MOL000546	Diosgenin	414.69	80.88	0.81
MOL002058	Medioresinol	388.45	57.20	0.62
MOL007356	Solanocapsine	430.75	52.94	0.67
MOL000098	Quercetin	302.25	46.43	0.28
MOL000953	Cholesterol	386.73	37.87	0.68
MOL002773	Beta-carotene	536.96	37.18	0.58
MOL000359	Sitosterol	414.79	36.91	0.75
MOL006853	Solasonine	884.19	25.94	0.06
MOL006851	Solamargine	577.89	10.90	0.29

**Table 2:** IC50 values of the SN extract and its sub-fractions in human ovarian cancer and normal epithelial cell lines.

Sample	IC50 Values ^1^		
	SKOV3	A2780	IOSE80PC
SN ex.	>200	111.50 ± 2.94	>200
n-hexane fr.	>200	135.61 ± 2.30	>200
CH_2_Cl_2_ fr. (SNMC)	>200	102.98 ± 3.11	>200
EtOAc fr.	168.39 ± 3.78	172.53 ± 5.48	>200
*n*-BuOH fr. (SNBT)	34.36 ± 0.06	92.09 ± 4.12	>200
Aqueous fr.	>200	>200	>200
Cisplatin	18.62 ± 1.40	10.63 ± 1.05	18.21 ± 0.55

^1^ The half-maximal inhibitory concentration (IC50) was determined after 48 h of treatment using an MTT assay. Values for the SN extract and its five solvent sub-fractions are expressed in μg/mL, whereas values for the positive control, cisplatin, are expressed in μM. Data are presented as the mean ± standard deviation (SD).

## Data Availability

The original contributions presented in this study are included in the article/Supplementary Materials. Further inquiries can be directed to the corresponding author.

## Supplementary Materials

The following supporting information can be downloaded at: https://www.mdpi.com/article/10.3390/ijms27167076/s1.

## Abbreviations

The following abbreviations are used in this manuscript:

AKT	Protein kinase B
ATCC	American Type Culture Collection
BAX	BCL2-associated X protein
BCL2	B-cell lymphoma 2
CASP3	Caspase-3
CASP8	Caspase-8
CASP9	Caspase-9
CAV1	Caveolin 1
CCND1	Cyclin D1
CD163	CD163 molecule
DAVID	Database for Annotation, Visualization and Integrated Discovery
DC	Degree Centrality
DL	Drug-likeness
ELISA	Enzyme-linked immunosorbent assay
EMT	Epithelial–mesenchymal transition
F3	Coagulation factor III, tissue factor
FAK	Focal adhesion kinase
GO	Gene Ontology
GO-BP	Gene Ontology Biological Process
GO-CC	Gene Ontology Cellular Component
GO-MF	Gene Ontology Molecular Function
HERB	A high-throughput experiment- and reference-guided database of traditional Chinese medicine
HIF1A	Hypoxia inducible factor 1 subunit alpha
HPLC	High-performance liquid chromatography
IC_50_	Half maximal inhibitory concentration
IL6	Interleukin 6
IκB	Inhibitor of nuclear factor kappa B
JUN	Jun proto-oncogene, AP-1 transcription factor subunit
KEGG	Kyoto Encyclopedia of Genes and Genomes
M2	M2 macrophage
MMP2	Matrix metallopeptidase 2
MMP9	Matrix metallopeptidase 9
MTT	3-(4,5-Dimethylthiazol-2-yl)-2,5-diphenyltetrazolium bromide
NF-κB	Nuclear factor kappa B
OB	Oral bioavailability
OVCA	Ovarian cancer
p65	Nuclear factor kappa B p65
PARP	Poly(ADP-ribose) polymerase
PCR	Polymerase chain reaction
PI	Propidium iodide
PI3K	Phosphoinositide 3-kinase
PPI	Protein–protein interaction
PRKCA	Protein kinase C alpha
PVDF	Polyvinylidene fluoride
RCSB PDB	Research Collaboratory for Structural Bioinformatics Protein Data Bank
RELA	RELA proto-oncogene, NF-κB subunit
RT	Retention time
SD	Standard deviation
SDS-PAGE	Sodium dodecyl sulfate–polyacrylamide gel electrophoresis
SMILES	Simplified Molecular Input Line Entry System
SN	*Solanum nigrum* L.
SNBT	Butanol fraction of *Solanum nigrum* L.
SNMC	Methylene chloride fraction of *Solanum nigrum* L.
STRING	Search Tool for the Retrieval of Interacting Genes/Proteins
TAMs	Tumor-associated macrophages
TBS-T	Tris-buffered saline with Tween 20
TCMSP	Traditional Chinese Medicine Systems Pharmacology Database and Analysis Platform
TGFB1	Transforming growth factor beta 1
TME	Tumor microenvironment
VEGF	Vascular endothelial growth factor
VEGFA	Vascular endothelial growth factor A
