# Supplementary material for: Unveiling the Dual Mechanisms of Solanum nigrum L. Fractions Against Ovarian Cancer via AKT/NF-κB and Tumor Microenvironment Modulation: An In Silico and In Vitro Study
Source: Int J Mol Sci. 2026 Aug 7;27(16):7076. doi: 10.3390/ijms27167076 (PMC13512683; doi:10.3390/ijms27167076)
Supplement: Supplementary file 1 [file ijms-27-07076-s001.zip › ijms-4462195-supplementary.pdf]

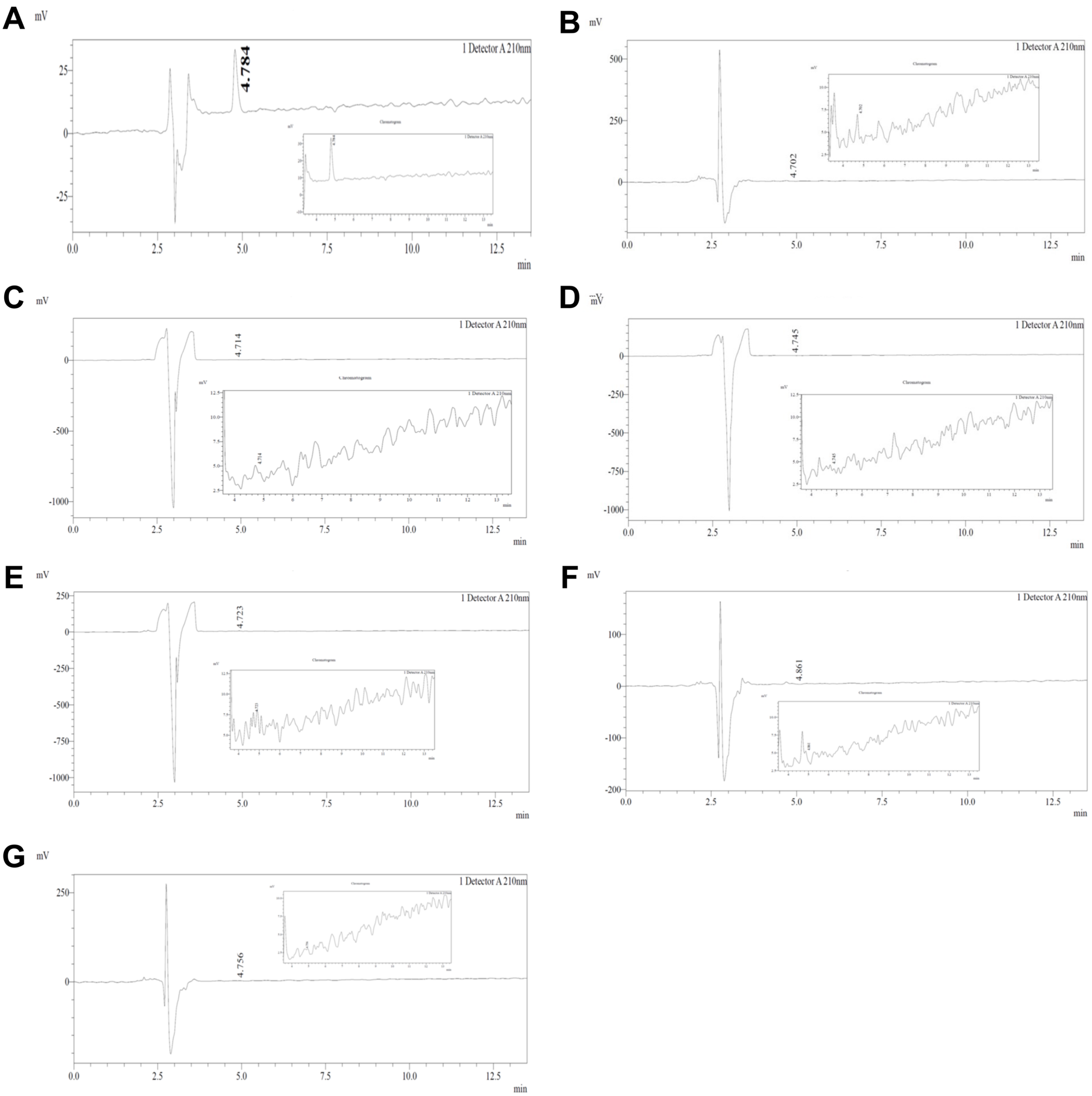

Figure S1. HPLC analysis of solasonine in SN extract and its fractions. All samples and solasonine standards were dissolved in acetonitrile: distilled water (1:1, v/v), filtered through a 0.22 μm syringe filter (Thermo Scientific, MA, USA), and analyzed using a Nexera-3 HPLC system (Shimadzu, Tokyo, Japan) equipped with a UV detector (SPD-40), degassing unit (DGU-403), solvent pump (LC-40BX3), system controller (SCL-40), and column oven (CTO-40S). Separation was performed on a TSKgel ODS-100V C18 column (250 mm × 4.6 mm, 5 μm; Tokyo, Japan). The mobile phases were A water with 0.1% formic acid and B acetonitrile with 0.1% formic acid. Elution was carried out at 1.0 mL/min with a gradient from 30% to 35% B over 11 min, and 35% to 45% B over the next 2 min. Detection was performed at 210 nm, with the column temperature set at 35 °C. A solasonine calibration curve was generated using serial dilutions of the standard. Retention times (RT) were as follows: (A) Standard (4.784 min), (B) SN extract (4.702 min), (C) *n*-hexane fraction (4.714 min), (D) methyl chloride fraction (4.745 min), (E) ethyl acetate fraction (4.723 min), (F) *n*-butanol fraction (4.861 min), and (G) aqueous fraction (4.756 min).

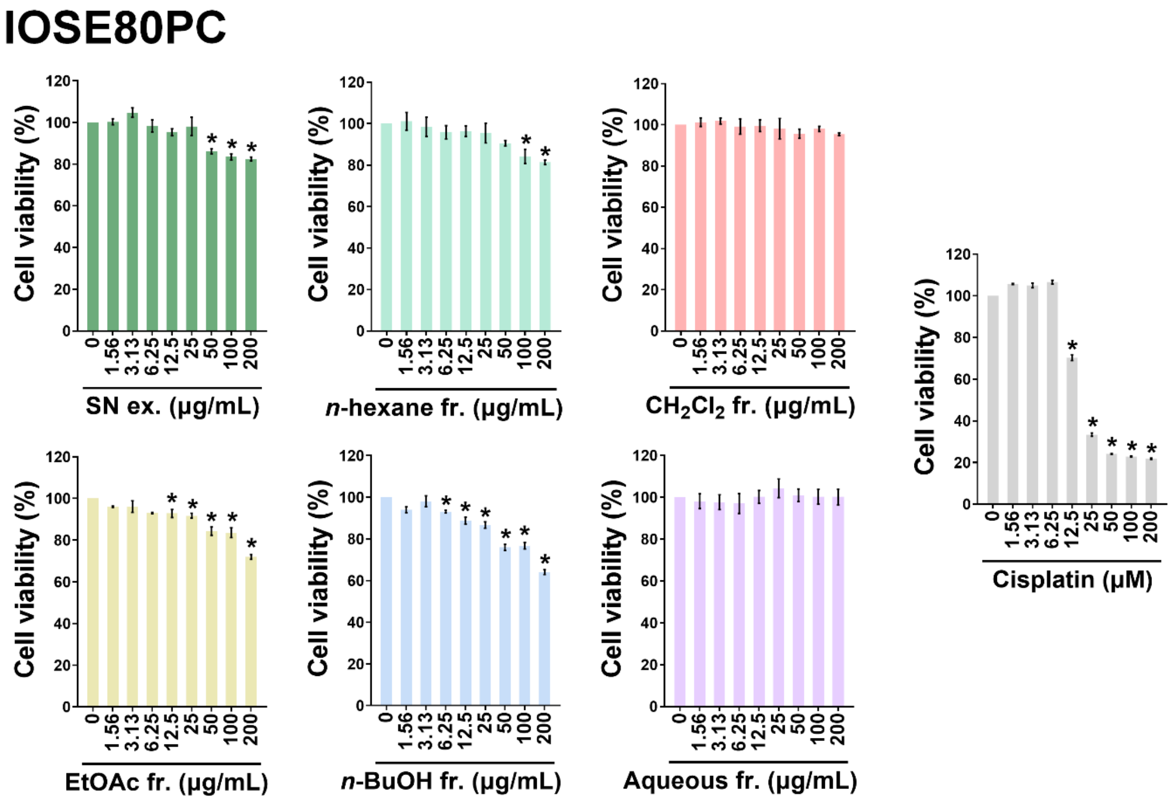

Figure S2. Cytotoxicity of Solanum nigrum extract, its sub-fractions, and cisplatin in IOSE80PC normal ovarian epithelial cells. Cells were treated with indicated concentrations of SN extract, its five solvent sub-fractions (0–200 μg/mL), or cisplatin (0–200 μM) for 48 h. Cell viability was assessed via MTT assay and expressed as a percentage relative to the untreated control. Data are presented as mean ± SD of two independent biological experiments (n = 2), each performed in technical triplicate. * $p < 0.05$ vs. the untreated control group.

Table S1. Comprehensive list of the 669 target genes associated with OVCA.

Gene Symbol	Description	Uniprot ID	Relevance score
BRCA1	BRCA1 DNA Repair Associated	P38398	631.30
BRCA2	BRCA2 DNA Repair Associated	P51587	560.70
TP53	Tumor Protein P53	P04637	262.98
ATM	ATM Serine/Threonine Kinase	Q13315	256.62
PALB2	Partner And Localizer Of BRCA2	Q86YC2	231.54
MSH6	MutS Homolog 6	P52701	226.31
CDH1	Cadherin 1	P12830	223.33
BRIP1	BRCA1 Interacting Helicase 1	Q9BX63	218.45
MSH2	MutS Homolog 2	P43246	217.98
CHEK2	Checkpoint Kinase 2	O96017	213.00
MLH1	MutL Homolog 1	P40692	205.16
APC	APC Regulator Of WNT Signaling Pathway	P25054	189.95
RAD51D	RAD51 Paralog D	O75771	185.79
PMS2	PMS1 Homolog 2, Mismatch Repair System Component	P54278	184.99
BARD1	BRCA1 Associated RING Domain 1	Q99728	180.09
PTEN	Phosphatase And Tensin Homolog	P60484	178.59
C11orf65	Chromosome 11 Open Reading Frame 65	Q8NCR3	166.95
EGFR	Epidermal Growth Factor Receptor	P00533	163.37
RAD51C	RAD51 Paralog C	O43502	163.08
POLE	DNA Polymerase Epsilon, Catalytic Subunit	Q07864	156.65
NBN	Nibrin	O60934	153.09
RAD50	RAD50 Double Strand Break Repair Protein	Q92878	148.32
PIK3CA	Phosphatidylinositol-4,5-Bisphosphate 3-Kinase Catalytic Subunit Alpha	P42336	144.32
DICER1	Dicer 1, Ribonuclease III	Q9UPY3	143.84
CDKN2A	Cyclin Dependent Kinase Inhibitor 2A	Q8N726	138.66
NF1	Neurofibromin 1	P21359	136.70
ERBB2	Erb-B2 Receptor Tyrosine Kinase 2	P04626	136.52
KRAS	KRAS Proto-Oncogene, GTPase	P01116	136.14
AXIN2	Axin 2	Q9Y2T1	135.35
MUTYH	MutY DNA Glycosylase	Q9UIF7	129.62
BRAF	B-Raf Proto-Oncogene, Serine/Threonine Kinase	P15056	126.73
BLM	BLM RecQ Like Helicase	P54132	126.47
RET	Ret Proto-Oncogene	P07949	125.86
MET	MET Proto-Oncogene, Receptor Tyrosine Kinase	P08581	125.86
TSC2	TSC Complex Subunit 2	P49815	123.49
CTNNB1	Catenin Beta 1	P35222	122.48
ALK	ALK Receptor Tyrosine Kinase	Q9UM73	122.21
POLD1	DNA Polymerase Delta 1, Catalytic Subunit	P28340	120.14
MLH3	MutL Homolog 3	Q9UHC1	119.73
MRE11	MRE11 Homolog, Double Strand Break Repair Nuclease	P49959	119.66
PTCH1	Patched 1	Q13635	119.25
RB1	RB Transcriptional Corepressor 1	P06400	118.62

AKT1	AKT Serine/Threonine Kinase 1	P31749	114.46
STK11	Serine/Threonine Kinase 11	Q15831	114.39
SMAD4	SMAD Family Member 4	Q13485	106.10
KIT	KIT Proto-Oncogene, Receptor Tyrosine Kinase	P10721	105.85
BMPR1A	Bone Morphogenetic Protein Receptor Type 1A	P36894	99.65
AR	Androgen Receptor	P10275	99.58
TSC1	TSC Complex Subunit 1	Q92574	99.26
SMARCA4	SWI/SNF Related, Matrix Associated, Actin Dependent Regulator Of Chromatin, Subfamily A, Member 4	P51532	99.20
FANCC	FA Complementation Group C	Q00597	99.00
CDKN1B	Cyclin Dependent Kinase Inhibitor 1B	P46527	98.02
TERT	Telomerase Reverse Transcriptase	O14746	94.95
PDGFRA	Platelet Derived Growth Factor Receptor Alpha	P16234	94.32
CTNNA1	Catenin Alpha 1	P35221	93.98
FH	Fumarate Hydratase	P07954	93.48
FLCN	Folliculin	Q8NFG4	92.79
MSH3	MutS Homolog 3	P20585	91.36
FANCM	FA Complementation Group M	Q8IYD8	89.36
ESR1	Estrogen Receptor 1	P03372	87.79
KIF1B	Kinesin Family Member 1B	O60333	84.37
EPCAM	Epithelial Cell Adhesion Molecule	P16422	82.51
XRCC2	X-Ray Repair Cross Complementing 2	O43543	81.92
AOPEP	Aminopeptidase O (Putative)	Q8N6M6	81.71
HRAS	HRas Proto-Oncogene, GTPase	P01112	79.93
CDK4	Cyclin Dependent Kinase 4	P11802	78.54
WT1	WT1 Transcription Factor	P19544	78.11
ERCC2	ERCC Excision Repair 2, TFIIH Core Complex Helicase Subunit	P18074	77.26
RECQL4	RecQ Like Helicase 4	O94761	75.34
FGFR2	Fibroblast Growth Factor Receptor 2	P21802	74.61
FANCD2	FA Complementation Group D2	Q9BXW9	74.50
TUG1	Taurine Up-Regulated 1	A0A6I8PU40	73.41
PMS1	PMS1 Homolog 1, Mismatch Repair System Component	P54277	72.74
SMARCB1	SWI/SNF Related, Matrix Associated, Actin Dependent Regulator Of Chromatin, Subfamily B, Member 1	Q12824	70.80
CCND1	Cyclin D1	P24385	70.61
RAD51	RAD51 Recombinase	Q06609	70.26
MTOR	Mechanistic Target Of Rapamycin Kinase	P42345	69.59
MYC	MYC Proto-Oncogene, BHLH Transcription Factor	P01106	68.03
ERCC4	ERCC Excision Repair 4, Endonuclease Catalytic Subunit	Q92889	67.69
ERCC5	ERCC Excision Repair 5, Endonuclease	P28715	67.15
FANCA	FA Complementation Group A	O15360	66.49
BUB1B	BUB1 Mitotic Checkpoint Serine/Threonine Kinase B	O60566	66.00
PRKN	Parkin RBR E3 Ubiquitin Protein Ligase	O60260	65.62
VHL	Von Hippel-Lindau Tumor Suppressor	P40337	65.58
RNASEL	Ribonuclease L	Q05823	65.20
WRN	WRN RecQ Like Helicase	Q14191	64.95

FSHR	Follicle Stimulating Hormone Receptor	P23945	64.42
MSR1	Macrophage Scavenger Receptor 1	P21757	63.45
CDKN2B	Cyclin Dependent Kinase Inhibitor 2B	P42772	63.16
HNF1B	HNF1 Homeobox B	P35680	62.80
ERBB3	Erb-B2 Receptor Tyrosine Kinase 3	P21860	61.93
STAT3	Signal Transducer And Activator Of Transcription 3	P40763	61.53
TMEM127	Transmembrane Protein 127	O75204	61.48
TGFBR2	Transforming Growth Factor Beta Receptor 2	P37173	61.16
FANCE	FA Complementation Group E	Q9HB96	60.89
MT-CYB	Mitochondrially Encoded Cytochrome B	P00156	60.78
CASP8	Caspase 8	Q14790	60.70
FANCG	FA Complementation Group G	O15287	60.37
PPM1D	Protein Phosphatase, Mg2+/Mn2+ Dependent 1D	O15297	59.72
CYP17A1	Cytochrome P450 Family 17 Subfamily A Member 1	P05093	59.24
MAP3K1	Mitogen-Activated Protein Kinase Kinase Kinase 1	Q13233	58.08
SDHAF2	Succinate Dehydrogenase Complex Assembly Factor 2	Q9NX18	57.99
BAX	BCL2 Associated X, Apoptosis Regulator	Q07812	57.97
ELAC2	ElaC Ribonuclease Z 2	Q9BQ52	57.91
NTRK1	Neurotrophic Receptor Tyrosine Kinase 1	P04629	57.88
CDKN1A	Cyclin Dependent Kinase Inhibitor 1A	P38936	57.73
ERCC3	ERCC Excision Repair 3, TFIIH Core Complex Helicase Subunit	P19447	57.66
XPC	XPC Complex Subunit, DNA Damage Recognition And Repair Factor	Q01831	57.17
VEGFA	Vascular Endothelial Growth Factor A	P15692	56.84
CYP19A1	Cytochrome P450 Family 19 Subfamily A Member 1	P11511	56.14
HMMR	Hyaluronan Mediated Motility Receptor	O75330	56.10
MDM2	MDM2 Proto-Oncogene	Q00987	54.73
XPA	XPA, DNA Damage Recognition And Repair Factor	P23025	54.62
ESR2	Estrogen Receptor 2	Q92731	54.57
HNF1A	HNF1 Homeobox A	P20823	54.47
EP300	E1A Binding Protein P300	Q09472	54.22
MAPK1	Mitogen-Activated Protein Kinase 1	P28482	53.86
BIVM-ERCC5	BIVM-ERCC5 Readthrough		53.51
NF2	NF2, Moesin-Ezrin-Radixin Like (MERLIN) Tumor Suppressor	P35240	53.31
RUNX1	RUNX Family Transcription Factor 1	Q01196	53.10
ATR	ATR Serine/Threonine Kinase	Q13535	53.00
IL1B	Interleukin 1 Beta	P01584	52.89
JUN	Jun Proto-Oncogene, AP-1 Transcription Factor Subunit	P05412	52.64
BCL2	BCL2 Apoptosis Regulator	P10415	52.62
XRCC3	X-Ray Repair Cross Complementing 3	O43542	52.37
MUC16	Mucin 16, Cell Surface Associated	Q8WXI7	52.33
HIF1A	Hypoxia Inducible Factor 1 Subunit Alpha	Q16665	51.88
TGFB1	Transforming Growth Factor Beta 1	P01137	51.55
FAS	Fas Cell Surface Death Receptor	P25445	51.49
TSHR	Thyroid Stimulating Hormone Receptor	P16473	51.39
ERCC1	ERCC Excision Repair 1, Endonuclease Non-Catalytic Subunit	P07992	50.77

RHOA	Ras Homolog Family Member A	P61586	50.61
FANCF	FA Complementation Group F	Q9NPI8	50.55
KDR	Kinase Insert Domain Receptor	P35968	50.09
IL6	Interleukin 6	P05231	50.04
MXI1	MAX Interactor 1, Dimerization Protein	P50539	49.90
FASLG	Fas Ligand	P48023	49.27
SRA1	Steroid Receptor RNA Activator 1	Q9HD15	49.26
IL2	Interleukin 2	P60568	48.66
AURKA	Aurora Kinase A	O14965	48.60
DHFR	Dihydrofolate Reductase	P00374	48.29
TNF	Tumor Necrosis Factor	P01375	48.12
PPARG	Peroxisome Proliferator Activated Receptor Gamma	P37231	48.11
IGF2	Insulin Like Growth Factor 2	P01344	48.02
PGR	Progesterone Receptor	P06401	47.93
TRIM24	Tripartite Motif Containing 24	O15164	47.51
SRC	SRC Proto-Oncogene, Non-Receptor Tyrosine Kinase	P12931	47.05
MMP2	Matrix Metallopeptidase 2	P08253	46.76
SMAD7	SMAD Family Member 7	O15105	46.66
RECK	Reversion Inducing Cysteine Rich Protein With Kazal Motifs	O95980	46.34
ABCB1	ATP Binding Cassette Subfamily B Member 1	P08183	46.08
PIK3R1	Phosphoinositide-3-Kinase Regulatory Subunit 1	P27986	45.59
CDK2	Cyclin Dependent Kinase 2	P24941	45.39
FANCD2OS	FANCD2 Opposite Strand	Q96PS1	45.13
EXT2	Exostosin Glycosyltransferase 2	Q93063	45.04
PTGS2	Prostaglandin-Endoperoxide Synthase 2	P35354	44.82
EXT1	Exostosin Glycosyltransferase 1	Q16394	44.81
ITGB1	Integrin Subunit Beta 1	P05556	44.78
OPCML	Opioid Binding Protein/Cell Adhesion Molecule Like	Q14982	44.54
MUC1	Mucin 1, Cell Surface Associated	P15941	44.36
RASSF1	Ras Association Domain Family Member 1	Q9NS23	44.22
RRAS2	RAS Related 2	P62070	44.22
JAK2	Janus Kinase 2	O60674	43.89
BIRC5	Baculoviral IAP Repeat Containing 5	O15392	43.52
CD274	CD274 Molecule	Q9NZQ7	43.38
PARP1	Poly(ADP-Ribose) Polymerase 1	P09874	43.20
MAP2K1	Mitogen-Activated Protein Kinase Kinase 1	Q02750	43.15
EGF	Epidermal Growth Factor	P01133	42.88
IGF1	Insulin Like Growth Factor 1	P05019	42.32
CASP3	Caspase 3	P42574	42.14
PPP2R1A	Protein Phosphatase 2 Scaffold Subunit Aalpha	P30153	41.55
MMP9	Matrix Metallopeptidase 9	P14780	41.48
GSTP1	Glutathione S-Transferase Pi 1	P09211	41.29
CXCL8	C-X-C Motif Chemokine Ligand 8	P10145	41.29
SOD2	Superoxide Dismutase 2	P04179	40.95
IRF1	Interferon Regulatory Factor 1	P10914	40.94

ZEB1	Zinc Finger E-Box Binding Homeobox 1	P37275	40.78
NRG1	Neuregulin 1	Q02297	40.51
ROCK1	Rho Associated Coiled-Coil Containing Protein Kinase 1	Q13464	40.47
ZNF276	Zinc Finger Protein 276	Q8N554	40.19
SHC1	SHC Adaptor Protein 1	P29353	39.78
WWOX	WW Domain Containing Oxidoreductase	Q9NZC7	39.74
PHB1	Prohibitin 1	P35232	39.61
AFP	Alpha Fetoprotein	P02771	39.42
FGFR1	Fibroblast Growth Factor Receptor 1	P11362	39.25
RAF1	Raf-1 Proto-Oncogene, Serine/Threonine Kinase	P04049	39.24
TYMS	Thymidylate Synthetase	P04818	39.18
BMP6	Bone Morphogenetic Protein 6	P22004	39.14
NFKB1	Nuclear Factor Kappa B Subunit 1	P19838	38.19
ABCG2	ATP Binding Cassette Subfamily G Member 2 (JR Blood Group)	Q9UNQ0	38.19
CD44	CD44 Molecule (IN Blood Group)	P16070	38.15
XRCC1	X-Ray Repair Cross Complementing 1	P18887	38.12
DROSHA	Drosha Ribonuclease III	Q9NRR4	38.02
CREBBP	CREB Binding Protein	Q92793	37.96
CTAG1B	Cancer/Testis Antigen 1B	P78358	37.70
CEACAM5	CEA Cell Adhesion Molecule 5	P06731	37.56
IGF1R	Insulin Like Growth Factor 1 Receptor	P08069	37.16
EPHB2	EPH Receptor B2	P29323	37.10
THBS1	Thrombospondin 1	P07996	36.79
BCL2L1	BCL2 Like 1	Q07817	36.76
KLK3	Kallikrein Related Peptidase 3	P07288	36.61
CD82	CD82 Molecule	P27701	36.46
CXCR4	C-X-C Motif Chemokine Receptor 4	P61073	36.35
DNMT3A	DNA Methyltransferase 3 Alpha	Q9Y6K1	36.35
NOTCH1	Notch Receptor 1	P46531	36.33
CYP1A1	Cytochrome P450 Family 1 Subfamily A Member 1	P04798	36.16
FANCI	FA Complementation Group I	Q9NVI1	35.70
HOXA11	Homeobox A11	P31270	35.57
BUB1	BUB1 Mitotic Checkpoint Serine/Threonine Kinase	O43683	35.36
MMP1	Matrix Metallopeptidase 1	P03956	35.35
CAST	Calpastatin	P20810	35.28
AKT2	AKT Serine/Threonine Kinase 2	P31751	35.27
MECOM	MDS1 And EVI1 Complex Locus	Q03112	35.21
FLT1	Fms Related Receptor Tyrosine Kinase 1	P17948	35.06
PCNA	Proliferating Cell Nuclear Antigen	P12004	35.04
IGFBP3	Insulin Like Growth Factor Binding Protein 3	P17936	34.94
TNFRSF19	TNF Receptor Superfamily Member 19	Q9NS68	34.84
H1-3	H1.3 Linker Histone, Cluster Member	P16402	34.84
CARS2	Cysteinyl-TRNA Synthetase 2, Mitochondrial	Q9HA77	34.84
PTK2	Protein Tyrosine Kinase 2	Q05397	34.76
MKI67	Marker Of Proliferation Ki-67	P46013	34.60

IFNG	Interferon Gamma	P01579	34.52
KRT7	Keratin 7	P08729	34.39
PLAU	Plasminogen Activator, Urokinase	P00749	34.31
GNRH1	Gonadotropin Releasing Hormone 1	P01148	34.19
MGMT	O-6-Methylguanine-DNA Methyltransferase	P16455	34.17
PIK3CG	Phosphatidylinositol-4,5-Bisphosphate 3-Kinase Catalytic Subunit Gamma	P48736	34.05
KRT20	Keratin 20	P35900	34.04
LHCGR	Luteinizing Hormone/Choriogonadotropin Receptor	P22888	34.03
SPAAR	Small Regulatory Polypeptide Of Amino Acid Response	A0A1B0GV Q0	33.86
IGSF9B	Immunoglobulin Superfamily Member 9B	Q9UPX0	33.68
IL10	Interleukin 10	P22301	33.66
CDK1	Cyclin Dependent Kinase 1	P06493	33.60
ABCC1	ATP Binding Cassette Subfamily C Member 1 (ABCC1 Blood Group)	P33527	33.59
SMAD3	SMAD Family Member 3	P84022	33.49
VEGFC	Vascular Endothelial Growth Factor C	P49767	33.38
DNMT1	DNA Methyltransferase 1	P26358	33.34
TNFSF10	TNF Superfamily Member 10	P50591	33.23
LCP1	Lymphocyte Cytosolic Protein 1	P13796	33.17
INS	Insulin	P01308	33.15
TP53BP1	Tumor Protein P53 Binding Protein 1	Q12888	33.13
CXCL12	C-X-C Motif Chemokine Ligand 12	P48061	32.89
CSF3	Colony Stimulating Factor 3	P09919	32.79
FGF2	Fibroblast Growth Factor 2	P09038	32.78
NQO1	NAD(P)H Quinone Dehydrogenase 1	P15559	32.76
TP73	Tumor Protein P73	O15350	32.74
MAPK3	Mitogen-Activated Protein Kinase 3	P27361	32.71
E2F1	E2F Transcription Factor 1	Q01094	32.60
AMH	Anti-Mullerian Hormone	P03971	32.53
MSLN	Mesothelin	Q13421	32.50
ERBB4	Erb-B2 Receptor Tyrosine Kinase 4	Q15303	32.45
TNFRSF10 B	TNF Receptor Superfamily Member 10b	O14763	32.37
CASP9	Caspase 9	P55211	32.35
SERPINE1	Serpin Family E Member 1	P05121	32.30
LEP	Leptin	P41159	32.28
MMP14	Matrix Metallopeptidase 14	P50281	32.26
STAT1	Signal Transducer And Activator Of Transcription 1	P42224	32.16
CHEK1	Checkpoint Kinase 1	O14757	32.06
TOP2A	DNA Topoisomerase II Alpha	P11388	32.04
CCND2	Cyclin D2	P30279	31.93
VIM	Vimentin	P08670	31.83
TGFA	Transforming Growth Factor Alpha	P01135	31.81
DPYD	Dihydropyrimidine Dehydrogenase	Q12882	31.80
CDKN3	Cyclin Dependent Kinase Inhibitor 3	Q16667	31.67
TWIST1	Twist Family BHLH Transcription Factor 1	Q15672	31.42

PDCD1	Programmed Cell Death 1	Q15116	31.36
CCNE1	Cyclin E1	P24864	31.22
FOXO3	Forkhead Box O3	O43524	31.20
AXIN1	Axin 1	O15169	31.12
XIAP	X-Linked Inhibitor Of Apoptosis	P98170	31.11
HGF	Hepatocyte Growth Factor	P14210	30.92
SP1	Sp1 Transcription Factor	P08047	30.66
CDK6	Cyclin Dependent Kinase 6	Q00534	30.65
MMP7	Matrix Metallopeptidase 7	P09237	30.61
TGFBR1	Transforming Growth Factor Beta Receptor 1	P36897	30.54
ZEB2	Zinc Finger E-Box Binding Homeobox 2	O60315	30.43
CAV1	Caveolin 1	Q03135	30.43
TLR4	Toll Like Receptor 4	O00206	30.24
SPP1	Secreted Phosphoprotein 1	P10451	30.24
MAPK8	Mitogen-Activated Protein Kinase 8	P45983	30.23
RBBP8	RB Binding Protein 8, Endonuclease	Q99708	30.01
GLI1	GLI Family Zinc Finger 1	P08151	29.88
RAC1	Rac Family Small GTPase 1	P63000	29.81
CTLA4	Cytotoxic T-Lymphocyte Associated Protein 4	P16410	29.76
ABL1	ABL Proto-Oncogene 1, Non-Receptor Tyrosine Kinase	P00519	29.76
BMP4	Bone Morphogenetic Protein 4	P12644	29.73
SKP2	S-Phase Kinase Associated Protein 2	Q13309	29.72
CSF1R	Colony Stimulating Factor 1 Receptor	P07333	29.68
NCOA3	Nuclear Receptor Coactivator 3	Q9Y6Q9	29.65
FN1	Fibronectin 1	P02751	29.58
INHA	Inhibin Subunit Alpha	P05111	29.53
PDGFRB	Platelet Derived Growth Factor Receptor Beta	P09619	29.48
MCL1	MCL1 Apoptosis Regulator, BCL2 Family Member	Q07820	29.42
TYMP	Thymidine Phosphorylase	P19971	29.38
FOS	Fos Proto-Oncogene, AP-1 Transcription Factor Subunit	P01100	29.24
GPC3	Glypican 3	P51654	29.19
MAPK14	Mitogen-Activated Protein Kinase 14	Q16539	29.16
SLC2A1	Solute Carrier Family 2 Member 1	P11166	29.16
OGG1	8-Oxoguanine DNA Glycosylase	O15527	29.14
CCNB1	Cyclin B1	P14635	29.03
RELA	RELA Proto-Oncogene, NF-KB Subunit	Q04206	29.00
HSPB1	Heat Shock Protein Family B (Small) Member 1	P04792	28.99
CYP1B1	Cytochrome P450 Family 1 Subfamily B Member 1	Q16678	28.97
SNAI1	Snail Family Transcriptional Repressor 1	O95863	28.97
TFF1	Trefoil Factor 1	P04155	28.96
WFDC2	WAP Four-Disulfide Core Domain 2	Q14508	28.84
GSK3B	Glycogen Synthase Kinase 3 Beta	P49841	28.59
NME1	NME/NM23 Nucleoside Diphosphate Kinase 1	P15531	28.59
PLK1	Polo Like Kinase 1	P53350	28.53
TIMP1	TIMP Metallopeptidase Inhibitor 1	P01033	28.52

POU5F1	POU Class 5 Homeobox 1	Q01860	28.39
IL1A	Interleukin 1 Alpha	P01583	28.36
SNAI2	Snail Family Transcriptional Repressor 2	O43623	28.35
NFKBIA	NFKB Inhibitor Alpha	P25963	28.18
SETD2	SET Domain Containing 2, Histone Lysine Methyltransferase	Q9BYW2	28.06
CYCS	Cytochrome C, Somatic	P99999	27.86
TOP1	DNA Topoisomerase I	P11387	27.75
RPS6KB1	Ribosomal Protein S6 Kinase B1	P23443	27.74
PLAUR	Plasminogen Activator, Urokinase Receptor	Q03405	27.67
AREG	Amphiregulin	P15514	27.67
CCNA2	Cyclin A2	P20248	27.67
EPHA2	EPH Receptor A2	P29317	27.62
RARB	Retinoic Acid Receptor Beta	P10826	27.62
PIK3CB	Phosphatidylinositol-4,5-Bisphosphate 3-Kinase Catalytic Subunit Beta	P42338	27.58
HSP90AA1	Heat Shock Protein 90 Alpha Family Class A Member 1	P07900	27.58
APEX1	Apurinic/Apyrimidinic Endodeoxyribonuclease 1	P27695	27.51
CFLAR	CASP8 And FADD Like Apoptosis Regulator	O15519	27.44
INSR	Insulin Receptor	P06213	27.38
SMAD2	SMAD Family Member 2	Q15796	27.33
SPINK1	Serine Peptidase Inhibitor Kazal Type 1	P00995	27.31
E2F4	E2F Transcription Factor 4	Q16254	27.28
FASN	Fatty Acid Synthase	P49327	27.23
CCL2	C-C Motif Chemokine Ligand 2	P13500	27.15
CSF1	Colony Stimulating Factor 1	P09603	27.12
BCL2L11	BCL2 Like 11	O43521	27.06
NPM1	Nucleophosmin 1	P06748	27.04
CTSD	Cathepsin D	P07339	26.82
BMP2	Bone Morphogenetic Protein 2	P12643	26.75
TIMP2	TIMP Metallopeptidase Inhibitor 2	P16035	26.74
CYP3A4	Cytochrome P450 Family 3 Subfamily A Member 4	P08684	26.65
CSF2	Colony Stimulating Factor 2	P04141	26.59
AKT3	AKT Serine/Threonine Kinase 3	Q9Y243	26.59
IL4	Interleukin 4	P05112	26.56
TGFB2	Transforming Growth Factor Beta 2	P61812	26.44
IGFBP2	Insulin Like Growth Factor Binding Protein 2	P18065	26.38
ANXA5	Annexin A5	P08758	26.25
MYCN	MYCN Proto-Oncogene, BHLH Transcription Factor	P04198	26.20
DPH1	Diphthamide Biosynthesis 1	Q9BZG8	26.12
DNMT3B	DNA Methyltransferase 3 Beta	Q9UBC3	26.11
PROM1	Prominin 1	O43490	25.90
IFI27	Interferon Alpha Inducible Protein 27	P40305	25.90
LGALS3	Galectin 3	P17931	25.84
AHR	Aryl Hydrocarbon Receptor	P35869	25.84
VEGFD	Vascular Endothelial Growth Factor D	O43915	25.74
PRKCA	Protein Kinase C Alpha	P17252	25.73

PRKDC	Protein Kinase, DNA-Activated, Catalytic Subunit	P78527	25.71
CBS	Cystathionine Beta-Synthase	P35520	25.62
JAK1	Janus Kinase 1	P23458	25.43
CCND3	Cyclin D3	P30281	25.37
CDH2	Cadherin 2	P19022	25.36
GATA3	GATA Binding Protein 3	P23771	25.35
GPER1	G Protein-Coupled Estrogen Receptor 1	Q99527	25.35
BSG	Basigin (Ok Blood Group)	P35613	25.31
KRT8	Keratin 8	P05787	25.22
ITGAV	Integrin Subunit Alpha V	P06756	25.19
HMGB1	High Mobility Group Box 1	P09429	25.15
ELAVL1	ELAV Like RNA Binding Protein 1	Q15717	25.15
SPARC	Secreted Protein Acidic And Cysteine Rich	P09486	25.12
PAK1	P21 (RAC1) Activated Kinase 1	Q13153	25.05
HDAC9	Histone Deacetylase 9	Q9UKV0	25.03
TNFRSF10A	TNF Receptor Superfamily Member 10a	O00220	25.03
SERPINB5	Serpin Family B Member 5	P36952	25.03
TIMP3	TIMP Metallopeptidase Inhibitor 3	P35625	25.01
ARAF	A-Raf Proto-Oncogene, Serine/Threonine Kinase	P10398	24.98
KRT18	Keratin 18	P05783	24.97
MMP3	Matrix Metallopeptidase 3	P08254	24.88
PRKACA	Protein Kinase CAMP-Activated Catalytic Subunit Alpha	P17612	24.87
CXCL1	C-X-C Motif Chemokine Ligand 1	P09341	24.83
EBAG9	Estrogen Receptor Binding Site Associated Antigen 9	O00559	24.81
IRS1	Insulin Receptor Substrate 1	P35568	24.81
WNT5A	Wnt Family Member 5A	P41221	24.70
NRP1	Neuropilin 1	O14786	24.69
HSD17B1	Hydroxysteroid 17-Beta Dehydrogenase 1	P14061	24.64
ETS1	ETS Proto-Oncogene 1, Transcription Factor	P14921	24.56
RARA	Retinoic Acid Receptor Alpha	P10276	24.50
MUC4	Mucin 4, Cell Surface Associated	Q99102	24.48
ALDH1A1	Aldehyde Dehydrogenase 1 Family Member A1	P00352	24.42
BECN1	Beclin 1	Q14457	24.39
HBEGF	Heparin Binding EGF Like Growth Factor	Q99075	24.38
EZR	Ezrin	P15311	24.38
IL17A	Interleukin 17A	Q16552	24.33
GDF15	Growth Differentiation Factor 15	Q99988	24.30
YBX1	Y-Box Binding Protein 1	P67809	24.27
GAPDH	Glyceraldehyde-3-Phosphate Dehydrogenase	P04406	24.26
BAD	BCL2 Associated Agonist Of Cell Death	Q92934	24.25
ANGPT2	Angiopoietin 2	O15123	24.13
ABCC2	ATP Binding Cassette Subfamily C Member 2	Q92887	24.08
TUBB3	Tubulin Beta 3 Class III	Q13509	24.05
MVP	Major Vault Protein	Q14764	23.97
HSPA4	Heat Shock Protein Family A (Hsp70) Member 4	P34932	23.93

GJA1	Gap Junction Protein Alpha 1	P17302	23.81
GRP	Gastrin Releasing Peptide	P07492	23.79
KLK10	Kallikrein Related Peptidase 10	O43240	23.79
EPHB4	EPH Receptor B4	P54760	23.78
DAPK1	Death Associated Protein Kinase 1	P53355	23.78
GNRHR	Gonadotropin Releasing Hormone Receptor	P30968	23.77
IFNA1	Interferon Alpha 1	P01562	23.72
ANXA2	Annexin A2	P07355	23.69
CALB2	Calbindin 2	P22676	23.69
TEK	TEK Receptor Tyrosine Kinase	Q02763	23.65
GPR68	G Protein-Coupled Receptor 68	Q15743	23.64
MTA1	Metastasis Associated 1	Q13330	23.59
ENG	Endoglin	P17813	23.57
LGALS1	Galectin 1	P09382	23.55
ITGB3	Integrin Subunit Beta 3	P05106	23.51
CCNG1	Cyclin G1	P51959	23.47
PLCG1	Phospholipase C Gamma 1	P19174	23.41
FSCN1	Fascin Actin-Bundling Protein 1	Q16658	23.38
IL18	Interleukin 18	Q14116	23.37
KITLG	KIT Ligand	P21583	23.34
E2F3	E2F Transcription Factor 3	O00716	23.29
CDH3	Cadherin 3	P22223	23.28
ITGA6	Integrin Subunit Alpha 6	P23229	23.25
LDHA	Lactate Dehydrogenase A	P00338	23.24
CTSB	Cathepsin B	P07858	23.23
PTGS1	Prostaglandin-Endoperoxide Synthase 1	P23219	23.17
CLDN4	Claudin 4	O14493	23.09
CDC42	Cell Division Cycle 42	P60953	23.06
GRB2	Growth Factor Receptor Bound Protein 2	P62993	23.04
HMGA2	High Mobility Group AT-Hook 2	P52926	23.02
CASP7	Caspase 7	P55210	22.94
CLU	Clusterin	P10909	22.93
CA9	Carbonic Anhydrase 9	Q16790	22.89
MAP2K4	Mitogen-Activated Protein Kinase Kinase 4	P45985	22.88
FOLR1	Folate Receptor Alpha	P15328	22.79
FGF7	Fibroblast Growth Factor 7	P21781	22.79
CDH13	Cadherin 13	P55290	22.79
GADD45A	Growth Arrest And DNA Damage Inducible Alpha	P24522	22.77
EDN1	Endothelin 1	P05305	22.75
ITGA5	Integrin Subunit Alpha 5	P08648	22.68
ILK	Integrin Linked Kinase	Q13418	22.67
SNCG	Synuclein Gamma	O76070	22.60
CCN2	Cellular Communication Network Factor 2	P29279	22.56
XRCC6	X-Ray Repair Cross Complementing 6	P12956	22.50
FGF1	Fibroblast Growth Factor 1	P05230	22.38

PTK2B	Protein Tyrosine Kinase 2 Beta	Q14289	22.36
PXN	Paxillin	P49023	22.35
HMGA1	High Mobility Group AT-Hook 1	P17096	22.30
NOTCH3	Notch Receptor 3	Q9UM47	22.25
FANCL	FA Complementation Group L	Q9NW38	22.14
DIABLO	Diablo IAP-Binding Mitochondrial Protein	Q9NR28	22.14
IDO1	Indoleamine 2,3-Dioxygenase 1	P14902	22.06
CEACAM 3	CEA Cell Adhesion Molecule 3	P40198	22.05
DAB2	DAB Adaptor Protein 2	P98082	22.03
CDKN2C	Cyclin Dependent Kinase Inhibitor 2C	P42773	22.02
AMHR2	Anti-Mullerian Hormone Receptor Type 2	Q16671	22.00
GATA4	GATA Binding Protein 4	P43694	21.92
SULF1	Sulfatase 1	Q8IWU6	21.87
EIF4EBP1	Eukaryotic Translation Initiation Factor 4E Binding Protein 1	Q13541	21.87
MAGEA1	MAGE Family Member A1	P43355	21.81
ALPP	Alkaline Phosphatase, Placental	P05187	21.80
CCR7	C-C Motif Chemokine Receptor 7	P32248	21.78
CDC25A	Cell Division Cycle 25A	P30304	21.77
IL6R	Interleukin 6 Receptor	P08887	21.77
KLK6	Kallikrein Related Peptidase 6	Q92876	21.76
ETV4	ETS Variant Transcription Factor 4	P43268	21.75
CUL1	Cullin 1	Q13616	21.75
CLDN3	Claudin 3	O15551	21.71
DIRAS3	DIRAS Family GTPase 3	O95661	21.69
TUBB	Tubulin Beta Class I	P07437	21.67
HLA-G	Major Histocompatibility Complex, Class I, G	P17693	21.65
HPSE	Heparanase	Q9Y251	21.65
ST14	ST14 Transmembrane Serine Protease Matriptase	Q9Y5Y6	21.58
JUP	Junction Plakoglobin	P14923	21.51
TSG101	Tumor Susceptibility 101	Q99816	21.38
TXN	Thioredoxin	P10599	21.37
PRSS21	Serine Protease 21	Q9Y6M0	21.31
ITGB4	Integrin Subunit Beta 4	P16144	21.25
LCN2	Lipocalin 2	P80188	21.24
STAT5A	Signal Transducer And Activator Of Transcription 5A	P42229	21.24
LOX	Lysyl Oxidase	P28300	21.23
TKT	Transketolase	P29401	21.21
CD36	CD36 Molecule (CD36 Blood Group)	P16671	21.20
IL7	Interleukin 7	P13232	21.16
FAP	Fibroblast Activation Protein Alpha	Q12884	21.16
DUSP1	Dual Specificity Phosphatase 1	P28562	21.12
CDC25C	Cell Division Cycle 25C	P30307	21.08
EIF4E	Eukaryotic Translation Initiation Factor 4E	P06730	21.04
MAGEA3	MAGE Family Member A3	P43357	21.01
VTN	Vitronectin	P04004	20.94

CD40	CD40 Molecule	P25942	20.94
F3	Coagulation Factor III, Tissue Factor	P13726	20.85
RHOC	Ras Homolog Family Member C	P08134	20.85
TNFRSF1A	TNF Receptor Superfamily Member 1A	P19438	20.84
IL24	Interleukin 24	Q13007	20.80
APAF1	Apoptotic Peptidase Activating Factor 1	O14727	20.77
FGF3	Fibroblast Growth Factor 3	P11487	20.71
STMN1	Stathmin 1	P16949	20.65
RAD52	RAD52 Homolog, DNA Repair Protein	P43351	20.62
HSPD1	Heat Shock Protein Family D (Hsp60) Member 1	P10809	20.62
TGM2	Transglutaminase 2	P21980	20.60
FILIP1L	Filamin A Interacting Protein 1 Like	Q4L180	20.58
ITGA2	Integrin Subunit Alpha 2	P17301	20.54
ACACA	Acetyl-CoA Carboxylase Alpha	Q13085	20.46
PTTG1	PTTG1 Regulator Of Sister Chromatid Separation, Securin	O95997	20.46
TFRC	Transferrin Receptor	P02786	20.42
SERPINB2	Serpin Family B Member 2	P05120	20.39
HSPA8	Heat Shock Protein Family A (Hsp70) Member 8	P11142	20.32
CGB5	Chorionic Gonadotropin Subunit Beta 5	P0DN86	20.29
HIC1	HIC ZBTB Transcriptional Repressor 1	Q14526	20.25
FGF4	Fibroblast Growth Factor 4	P08620	20.24
DPP4	Dipeptidyl Peptidase 4	P27487	20.22
IGFBP5	Insulin Like Growth Factor Binding Protein 5	P24593	20.18
MYB	MYB Proto-Oncogene, Transcription Factor	P10242	20.15
TGFB3	Transforming Growth Factor Beta 3	P10600	20.14
EDNRA	Endothelin Receptor Type A	P25101	20.13
IL3	Interleukin 3	P08700	20.13
VTCN1	V-Set Domain Containing T Cell Activation Inhibitor 1	Q7Z7D3	20.10
CD34	CD34 Molecule	P28906	19.98
NANOG	Nanog Homeobox	Q9H9S0	19.94
NCOA4	Nuclear Receptor Coactivator 4	Q13772	19.91
H2AX	H2A.X Variant Histone	P16104	19.90
TFAP2A	Transcription Factor AP-2 Alpha	P05549	19.89
ANGPT1	Angiopoietin 1	Q15389	19.87
EMSY	EMSY Transcriptional Repressor, BRCA2 Interacting	Q7Z589	19.78
FBLN1	Fibulin 1	P23142	19.77
CD80	CD80 Molecule	P33681	19.76
ADAM17	ADAM Metallopeptidase Domain 17	P78536	19.69
CASP2	Caspase 2	P42575	19.63
JAK3	Janus Kinase 3	P52333	19.58
KLRK1	Killer Cell Lectin Like Receptor K1	P26718	19.57
HSPA1A	Heat Shock Protein Family A (Hsp70) Member 1A	P0DMV8	19.52
KLK8	Kallikrein Related Peptidase 8	O60259	19.51
MIF	Macrophage Migration Inhibitory Factor	P14174	19.49
CYP24A1	Cytochrome P450 Family 24 Subfamily A Member 1	Q07973	19.42

FGF8	Fibroblast Growth Factor 8	P55075	19.27
GSN	Gelsolin	P06396	19.26
NCOA1	Nuclear Receptor Coactivator 1	Q15788	19.25
S100A6	S100 Calcium Binding Protein A6	P06703	19.22
PPARA	Peroxisome Proliferator Activated Receptor Alpha	Q07869	19.20
GRPR	Gastrin Releasing Peptide Receptor	P30550	19.11
IGFBP4	Insulin Like Growth Factor Binding Protein 4	P22692	19.08
JAG1	Jagged Canonical Notch Ligand 1	P78504	19.07
F2R	Coagulation Factor II Thrombin Receptor	P25116	19.06
PECAM1	Platelet And Endothelial Cell Adhesion Molecule 1	P16284	19.04
LGALS3BP	Galectin 3 Binding Protein	Q08380	19.04
TNFRSF10D	TNF Receptor Superfamily Member 10d	Q9UBN6	19.02
IL11	Interleukin 11	P20809	19.01
CCNA1	Cyclin A1	P78396	19.01
NES	Nestin	P48681	18.98
HDAC6	Histone Deacetylase 6	Q9UBN7	18.91
LPAR1	Lysophosphatidic Acid Receptor 1	Q92633	18.83
E2F2	E2F Transcription Factor 2	Q14209	18.82
ATF3	Activating Transcription Factor 3	P18847	18.80
ABCC3	ATP Binding Cassette Subfamily C Member 3	O15438	18.79
LIF	LIF Interleukin 6 Family Cytokine	P15018	18.73
TACSTD2	Tumor Associated Calcium Signal Transducer 2	P09758	18.73
KLK4	Kallikrein Related Peptidase 4	Q9Y5K2	18.70
FST	Follistatin	P19883	18.70
KLK7	Kallikrein Related Peptidase 7	P49862	18.66
SELENBP1	Selenium Binding Protein 1	Q13228	18.57
CDK7	Cyclin Dependent Kinase 7	P50613	18.57
VCAN	Versican	P13611	18.56
CDH5	Cadherin 5	P33151	18.52
RRM2	Ribonucleotide Reductase Regulatory Subunit M2	P31350	18.52
HP	Haptoglobin	P00738	18.49
CASP1	Caspase 1	P29466	18.48
EIF2AK3	Eukaryotic Translation Initiation Factor 2 Alpha Kinase 3	Q9NZJ5	18.47
STC1	Stanniocalcin 1	P52823	18.40
HPN	Hepsin	P05981	18.32
ESRRA	Estrogen Related Receptor Alpha	P11474	18.30
GRN	Granulin Precursor	P28799	18.25
SFN	Stratifin	P31947	18.06
ESRRB	Estrogen Related Receptor Beta	O95718	17.99
CD86	CD86 Molecule	P42081	17.98
DCK	Deoxycytidine Kinase	P27707	17.93
CTSL	Cathepsin L	P07711	17.89
L1CAM	L1 Cell Adhesion Molecule	P32004	17.84
JUND	JunD Proto-Oncogene, AP-1 Transcription Factor Subunit	P17535	17.73
GATA6	GATA Binding Protein 6	Q92908	17.71

TPD52	Tumor Protein D52	P55327	17.61
TNFRSF10C	TNF Receptor Superfamily Member 10c	O14798	17.58
PTPN1	Protein Tyrosine Phosphatase Non-Receptor Type 1	P18031	17.55
PLG	Plasminogen	P00747	17.53
FGF9	Fibroblast Growth Factor 9	P31371	17.48
HNF4A	Hepatocyte Nuclear Factor 4 Alpha	P41235	17.45
SAT1	Spermidine/Spermine N1-Acetyltransferase 1	P21673	17.43
RAB25	RAB25, Member RAS Oncogene Family	P57735	17.38
DCN	Decorin	P07585	17.37
PARP2	Poly(ADP-Ribose) Polymerase 2	Q9UGN5	17.23
NACC1	Nucleus Accumbens Associated 1	Q96RE7	17.23
PTPN13	Protein Tyrosine Phosphatase Non-Receptor Type 13	Q12923	17.18
SLPI	Secretory Leukocyte Peptidase Inhibitor	P03973	17.06
CLEC3B	C-Type Lectin Domain Family 3 Member B	P05452	17.03
HELLS	Helicase, Lymphoid Specific	Q9NRZ9	16.99
IL4R	Interleukin 4 Receptor	P24394	16.97
THY1	Thy-1 Cell Surface Antigen	P04216	16.92
IL13RA2	Interleukin 13 Receptor Subunit Alpha 2	Q14627	16.92
NOTCH4	Notch Receptor 4	Q99466	16.92
NBR1	NBR1 Autophagy Cargo Receptor	Q14596	16.90
LPAR3	Lysophosphatidic Acid Receptor 3	Q9UBY5	16.85
ARNT	Aryl Hydrocarbon Receptor Nuclear Translocator	P27540	16.80
KLK5	Kallikrein Related Peptidase 5	Q9Y337	16.76
ENPP2	Ectonucleotide Pyrophosphatase/Phosphodiesterase 2	Q13822	16.68
TUBA1B	Tubulin Alpha 1b	P68363	16.60
ETS2	ETS Proto-Oncogene 2, Transcription Factor	P15036	16.50
SLC19A1	Solute Carrier Family 19 Member 1	P41440	16.49
CDC20	Cell Division Cycle 20	Q12834	16.49
CXADR	CXADR Ig-Like Cell Adhesion Molecule	P78310	16.45
SPINT1	Serine Peptidase Inhibitor, Kunitz Type 1	O43278	16.41
ZNF217	Zinc Finger Protein 217	O75362	16.39
GNRH2	Gonadotropin Releasing Hormone 2	O43555	16.29
AKR1C1	Aldo-Keto Reductase Family 1 Member C1	Q04828	16.28
LYVE1	Lymphatic Vessel Endothelial Hyaluronan Receptor 1	Q9Y5Y7	16.24
SPA17	Sperm Autoantigenic Protein 17	Q15506	16.24
GLS	Glutaminase	O94925	16.19
CD59	CD59 Molecule (CD59 Blood Group)	P13987	16.10
CD55	CD55 Molecule (Cromer Blood Group)	P08174	16.07
RBP1	Retinol Binding Protein 1	P09455	15.99
CD46	CD46 Molecule	P15529	15.93
LPAR2	Lysophosphatidic Acid Receptor 2	Q9HBW0	15.92
CSK	C-Terminal Src Kinase	P41240	15.90
ATP7B	ATPase Copper Transporting Beta	P35670	15.87
SCGB2A1	Secretoglobin Family 2A Member 1	O75556	15.79
RAN	RAN, Member RAS Oncogene Family	P62826	15.78

PYCARD	PYD And CARD Domain Containing	Q9ULZ3	15.75
ANXA3	Annexin A3	P12429	15.65
SERPINF1	Serpin Family F Member 1	P36955	15.59
ANG	Angiogenin	P03950	15.57
LGR6	Leucine Rich Repeat Containing G Protein-Coupled Receptor 6	Q9HBX8	15.52
HAS2	Hyaluronan Synthase 2	Q92819	15.50
AGTR1	Angiotensin II Receptor Type 1	P30556	15.25
PAEP	Progestagen Associated Endometrial Protein	P09466	15.22
TGFBI	Transforming Growth Factor Beta Induced	Q15582	15.22
SREBF2	Sterol Regulatory Element Binding Transcription Factor 2	Q12772	15.14
KLK13	Kallikrein Related Peptidase 13	Q9UKR3	15.13
S1PR1	Sphingosine-1-Phosphate Receptor 1	P21453	15.11
REL	REL Proto-Oncogene, NF-KB Subunit	Q04864	15.09
CD247	CD247 Molecule	P20963	15.07
CCBE1	Collagen And Calcium Binding EGF Domains 1	Q6UXH8	14.80
RSF1	Remodeling And Spacing Factor 1	Q96T23	14.76
TMPRSS3	Transmembrane Serine Protease 3	P57727	14.64
HAS1	Hyaluronan Synthase 1	Q92839	14.60
ETV7	ETS Variant Transcription Factor 7	Q9Y603	14.56
KLK14	Kallikrein Related Peptidase 14	Q9P0G3	14.56
CCR1	C-C Motif Chemokine Receptor 1	P32246	14.46
EIF5A	Eukaryotic Translation Initiation Factor 5A	P63241	14.41
ATP7A	ATPase Copper Transporting Alpha	Q04656	14.04
BCL2A1	BCL2 Related Protein A1	Q16548	13.99
FURIN	Furin, Paired Basic Amino Acid Cleaving Enzyme	P09958	13.88
CDR2	Cerebellar Degeneration Related Protein 2	Q01850	13.87
FGF5	Fibroblast Growth Factor 5	P12034	13.82
MAP3K5	Mitogen-Activated Protein Kinase Kinase Kinase 5	Q99683	13.78
PLAGL1	PLAG1 Like Zinc Finger 1	Q9UM63	13.76
LTBP1	Latent Transforming Growth Factor Beta Binding Protein 1	Q14766	13.76
DHDH	Dihydrodiol Dehydrogenase	Q9UQ10	13.74
PRSS8	Serine Protease 8	Q16651	13.72
AMBP	Alpha-1-Microglobulin/Bikunin Precursor	P02760	13.50
AKAP3	A-Kinase Anchoring Protein 3	O75969	13.50
SLC31A1	Solute Carrier Family 31 Member 1	O15431	13.48
HSPB2	Heat Shock Protein Family B (Small) Member 2	Q16082	13.48
RGCC	Regulator Of Cell Cycle	Q9H4X1	13.16
RNASET2	Ribonuclease T2	O00584	13.12
FGF13	Fibroblast Growth Factor 13	Q92913	12.83
ASNS	Asparagine Synthetase (Glutamine-Hydrolyzing)	P08243	12.83
ALPG	Alkaline Phosphatase, Germ Cell	P10696	12.73
OVGP1	Oviductal Glycoprotein 1	Q12889	12.44
HAS3	Hyaluronan Synthase 3	O00219	11.87
GPR4	G Protein-Coupled Receptor 4	P46093	10.76

Data were retrieved from the GeneCards (https://www.genecards.org/) and MalaCards (https://www.malacards.org/) databases using "ovarian cancer" as the keyword. The results were filtered exclusively for protein-coding genes to ensure therapeutic relevance.

Table S2. Comprehensive list of the 62 predicted target genes associated with SN.

Target name	Target ID	Ensembl ID
NR3C1	HBTAR001692	ENSG00000113580
PTGS2	HBTAR003295	ENSG00000073756
RTP1	HBTAR010938	ENSG00000175077
APP	HBTAR000220	ENSG00000142192
EDN1	HBTAR001160	ENSG00000078401
GCG	HBTAR001526	ENSG00000115263
HCRT	HBTAR001789	ENSG00000161610
ICAM1	HBTAR001976	ENSG00000090339
NOS2	HBTAR002737	ENSG00000007171
NOS3	HBTAR002738	ENSG00000164867
P2RY1	HBTAR002834	ENSG00000169860
PTGER3	HBTAR003287	ENSG00000050628
SLC5A7	HBTAR009040	ENSG00000115665
CASP1	HBTAR000515	ENSG00000137752
CASP3	HBTAR000517	ENSG00000164305
F3	HBTAR001305	ENSG00000117525
HIF1A	HBTAR001810	ENSG00000100644
IL6	HBTAR002055	ENSG00000136244
IL10	HBTAR002064	ENSG00000136634
MMP1	HBTAR002507	ENSG00000196611
MMP9	HBTAR002512	ENSG00000100985
TNF	HBTAR004140	ENSG00000232810
VEGFA	HBTAR004310	ENSG00000112715
COL7A1	HBTAR000791	ENSG00000114270
TGFB1	HBTAR004072	ENSG00000105329
RETN	HBTAR008621	ENSG00000104918
BCL2	HBTAR000360	ENSG00000171791
CASP8	HBTAR000522	ENSG00000064012
CASP9	HBTAR000523	ENSG00000132906
CAV1	HBTAR000529	ENSG00000105974
COX10	HBTAR000821	ENSG00000006695
GJA1	HBTAR001563	ENSG00000152661
JUN	HBTAR002166	ENSG00000177606
MMP2	HBTAR002508	ENSG00000087245
MMP10	HBTAR002513	ENSG00000166670
VEZF1	HBTAR004450	ENSG00000136451
TCEAL7	HBTAR008627	ENSG00000182916
Tstap198-15	HBTAR010531	None
Gja6	HBTAR012319	None
Ptges3-ps	HBTAR012515	None
Api5-ps	HBTAR012553	None
BAX	HBTAR000351	ENSG00000087088
MAP2	HBTAR002406	ENSG00000078018

PON1	HBTAR003098	ENSG00000005421
PRKCA	HBTAR003177	ENSG00000154229
CCND1	HBTAR000359	ENSG00000110092
CAT	HBTAR000528	ENSG00000121691
RELA	HBTAR003434	ENSG00000173039
SOD1	HBTAR003849	ENSG00000142168
CREG1	HBTAR004876	ENSG00000143162
EBAG9	HBTAR005073	ENSG00000147654
Sod1m	HBTAR010530	None
Appbp2os	HBTAR012453	None
DUOX2	HBTAR007419	ENSG00000140279
NCF1	HBTAR012441	ENSG00000158517
TP53COR1	HBTAR012603	None
ERBB2	HBTAR001263	ENSG00000141736
GAP43	HBTAR001503	ENSG00000172020
SCD	HBTAR003621	ENSG00000099194
Ciaaq1	HBTAR012518	None
MGAM	HBTAR004966	ENSG00000257335
CD40LG	HBTAR000592	ENSG00000102245

Target genes associated with SN were collected from the HERB database (http://herb.ac.cn/).

Table S3. Comprehensive list of the 189 predicted target genes associated with the 9 active compounds of SN.

Compounds	Ingredient ID	Target name	Target ID	Ensembl ID	Count
Diosgenin	HBIN024164	AKT1	HBTAR000130	ENSG00000142208	26
		CAT	HBTAR000528	ENSG00000121691	
		CDKN1A	HBTAR000641	ENSG00000124762	
		ABCC2	HBTAR000763	ENSG00000023839	
		ESR1	HBTAR001277	ENSG00000091831	
		FASN	HBTAR001337	ENSG00000169710	
		MTOR	HBTAR001437	ENSG00000198793	
		HIF1A	HBTAR001810	ENSG00000100644	
		NR3C2	HBTAR002504	ENSG00000151623	
		PGR	HBTAR002971	ENSG00000082175	
		PLA2G4A	HBTAR003023	ENSG00000116711	
		PTGS2	HBTAR003295	ENSG00000073756	
		RELA	HBTAR003434	ENSG00000173039	
		SOD1	HBTAR003849	ENSG00000142168	
		TP53	HBTAR004157	ENSG00000141510	
		VEGFA	HBTAR004310	ENSG00000112715	
		NR1I2	HBTAR004913	ENSG00000144852	
		NR3C1	HBTAR001692	ENSG00000113580	
		AR	HBTAR000233	ENSG00000169083	
		RPL17-C18orf32	HBTAR012911	ENSG00000215472	
		RPL17	HBTAR003512	ENSG00000265681	
		PDCD	HBTAR002897	None	
		TNFRSF10B	HBTAR004868	ENSG00000120889	
		MAPK3	HBTAR003193	ENSG00000102882	
		MAPK14	HBTAR000874	ENSG00000112062	
		MAPK1	HBTAR003192	ENSG00000100030	
Medioresinol	HBIN009785	KCNH2	HBTAR002192	ENSG00000055118	20
		PIM1	HBTAR003005	ENSG00000137193	
		ESR1	HBTAR001277	ENSG00000091831	
		NOS2	HBTAR002737	ENSG00000007171	
		GSK3B	HBTAR001710	ENSG00000082701	
		SCN5A	HBTAR003629	ENSG00000183873	
		CCNA2	HBTAR000549	ENSG00000145386	
		NCOA2	HBTAR005869	ENSG00000140396	
		ESR2	HBTAR001278	ENSG00000140009	
		ACHE	HBTAR000031	ENSG00000087085	
		CALM3	HBTAR000492	ENSG00000160014	
		DPP4	HBTAR001096	ENSG00000197635	
		PTGS1	HBTAR003294	ENSG00000095303	
		PTGS2	HBTAR003295	ENSG00000073756	
		PPARG	HBTAR003112	ENSG00000132170	

		ADRA1B	HBTAR000093	ENSG00000170214	
		F7	HBTAR001307	ENSG00000057593	
		AR	HBTAR000233	ENSG00000169083	
		CA2	HBTAR000460	ENSG00000104267	
		PRSS1	HBTAR003223	ENSG00000204983	
Solanocapsine	HBIN044305	AR	HBTAR000233	ENSG00000169083	1
Quercetin	HBIN041495	ACACA	HBTAR000021	ENSG00000278540	161
		ACHE	HBTAR000031	ENSG00000087085	
		ACP3	HBTAR000040	ENSG00000014257	
		PARP1	HBTAR000090	ENSG00000143799	
		ADRB2	HBTAR000099	ENSG00000169252	
		AHR	HBTAR000124	ENSG00000106546	
		AKT1	HBTAR000130	ENSG00000142208	
		AKR1B1	HBTAR000148	ENSG00000085662	
		ALOX5	HBTAR000151	ENSG00000012779	
		BIRC5	HBTAR000207	ENSG00000089685	
		AR	HBTAR000233	ENSG00000169083	
		BAX	HBTAR000351	ENSG00000087088	
		CCND1	HBTAR000359	ENSG00000110092	
		BCL2	HBTAR000360	ENSG00000171791	
		BCL2L1	HBTAR000362	ENSG00000171552	
		BMP2	HBTAR000392	ENSG00000125845	
		CASP3	HBTAR000517	ENSG00000164305	
		CASP8	HBTAR000522	ENSG00000064012	
		CASP9	HBTAR000523	ENSG00000132906	
		CAT	HBTAR000528	ENSG00000121691	
		CAV1	HBTAR000529	ENSG00000105974	
		RUNX2	HBTAR000531	ENSG00000124813	
		RUNX1T1	HBTAR000533	ENSG00000079102	
		CCNB1	HBTAR000550	ENSG00000134057	
		CD40LG	HBTAR000592	ENSG00000102245	
		CDKN1A	HBTAR000641	ENSG00000124762	
		CDKN2A	HBTAR000644	ENSG00000147889	
		CHUK	HBTAR000718	ENSG00000213341	
		COL1A1	HBTAR000776	ENSG00000108821	
		COL3A1	HBTAR000779	ENSG00000168542	
		CLDN4	HBTAR000831	ENSG00000189143	
		CRP	HBTAR000862	ENSG00000132693	
		CTSD	HBTAR000927	ENSG00000117984	
		CYP1A1	HBTAR000948	ENSG00000140465	
		CYP1A2	HBTAR000949	ENSG00000140505	
		CYP1B1	HBTAR000950	ENSG00000138061	
		CYP3A4	HBTAR000964	ENSG00000160868	
		NQO1	HBTAR001048	ENSG00000181019	
		DIO1	HBTAR001051	ENSG00000211452	

E2F1	HBTAR001141	ENSG00000101412
E2F2	HBTAR001142	ENSG00000007968
EGF	HBTAR001179	ENSG00000138798
EGFR	HBTAR001185	ENSG00000146648
ELK1	HBTAR001215	ENSG00000126767
ERBB2	HBTAR001263	ENSG00000141736
ERBB3	HBTAR001264	ENSG00000065361
ESR1	HBTAR001277	ENSG00000091831
ESR2	HBTAR001278	ENSG00000140009
F3	HBTAR001305	ENSG00000117525
FOS	HBTAR001429	ENSG00000170345
GJA1	HBTAR001563	ENSG00000152661
CXCL2	HBTAR001703	ENSG00000081041
GSK3B	HBTAR001710	ENSG00000082701
GSTM1	HBTAR001719	ENSG00000134184
GSTM2	HBTAR001720	ENSG00000213366
GSTP1	HBTAR001724	ENSG00000084207
HAS2	HBTAR001775	ENSG00000170961
HIF1A	HBTAR001810	ENSG00000100644
HK2	HBTAR001817	ENSG00000159399
HMOX1	HBTAR001851	ENSG00000100292
HSF1	HBTAR001932	ENSG00000185122
HSPA5	HBTAR001941	ENSG00000044574
HSPB1	HBTAR001945	ENSG00000106211
ICAM1	HBTAR001976	ENSG00000090339
IFNG	HBTAR002013	ENSG00000111537
IGF2	HBTAR002019	ENSG00000167244
IGFBP3	HBTAR002024	ENSG00000146674
IL1A	HBTAR002041	ENSG00000115008
IL1B	HBTAR002042	ENSG00000125538
IL2	HBTAR002045	ENSG00000109471
IL6	HBTAR002055	ENSG00000136244
CXCL8	HBTAR002060	ENSG00000169429
IL10	HBTAR002064	ENSG00000136634
CXCL10	HBTAR002097	ENSG00000169245
INSR	HBTAR002108	ENSG00000171105
IRF1	HBTAR002116	ENSG00000125347
EIF6	HBTAR002142	ENSG00000242372
JUN	HBTAR002166	ENSG00000177606
KCNH2	HBTAR002192	ENSG00000055118
MAOB	HBTAR002403	ENSG00000069535
MMP1	HBTAR002507	ENSG00000196611
MMP2	HBTAR002508	ENSG00000087245
MMP3	HBTAR002509	ENSG00000149968
MMP9	HBTAR002512	ENSG00000100985

MPO	HBTAR002535	ENSG00000005381
MYC	HBTAR002597	ENSG00000136997
NFE2L2	HBTAR002700	ENSG00000116044
NFKBIA	HBTAR002707	ENSG00000100906
NKX3-1	HBTAR002723	ENSG00000167034
NOS2	HBTAR002737	ENSG00000007171
NOS3	HBTAR002738	ENSG00000164867
ODC1	HBTAR002800	ENSG00000115758
SERPINE1	HBTAR002850	ENSG00000106366
PCOLCE	HBTAR002887	ENSG00000106333
PLAT	HBTAR003028	ENSG00000104368
PLAU	HBTAR003029	ENSG00000122861
POLD1	HBTAR003080	ENSG00000062822
PON1	HBTAR003098	ENSG00000005421
POR	HBTAR003101	ENSG00000127948
PPARA	HBTAR003110	ENSG00000186951
PPARD	HBTAR003111	ENSG00000112033
PPARG	HBTAR003112	ENSG00000132170
PRKCA	HBTAR003177	ENSG00000154229
PRKCB	HBTAR003178	ENSG00000166501
MAPK1	HBTAR003192	ENSG00000100030
MAPK3	HBTAR003193	ENSG00000102882
PRSS1	HBTAR003223	ENSG00000204983
PSMD3	HBTAR003265	ENSG00000108344
PTEN	HBTAR003282	ENSG00000171862
PTGER3	HBTAR003287	ENSG00000050628
PTGS1	HBTAR003294	ENSG00000095303
PTGS2	HBTAR003295	ENSG00000073756
RAF1	HBTAR003382	ENSG00000132155
RASA1	HBTAR003404	ENSG00000145715
RB1	HBTAR003408	ENSG00000139687
RELA	HBTAR003434	ENSG00000173039
RXRA	HBTAR003583	ENSG00000186350
SCN5A	HBTAR003629	ENSG00000183873
CCL2	HBTAR003641	ENSG00000108691
CXCL11	HBTAR003658	ENSG00000169248
SELE	HBTAR003676	ENSG00000007908
SLC2A4	HBTAR003749	ENSG00000181856
SOD1	HBTAR003849	ENSG00000142168
SOD3	HBTAR003851	ENSG00000109610
SPP1	HBTAR003881	ENSG00000118785
STAT1	HBTAR003926	ENSG00000115415
SULT1E1	HBTAR003936	ENSG00000109193
TGFB1	HBTAR004072	ENSG00000105329
THBD	HBTAR004087	ENSG00000178726

		TNF	HBTAR004140	ENSG00000232810	
		TOP1	HBTAR004153	ENSG00000198900	
		TOP2A	HBTAR004154	ENSG00000131747	
		TP53	HBTAR004157	ENSG00000141510	
		VCAM1	HBTAR004304	ENSG00000162692	
		VEGFA	HBTAR004310	ENSG00000112715	
		XDH	HBTAR004356	ENSG00000158125	
		DCAF5	HBTAR004883	ENSG00000139990	
		NR1I2	HBTAR004913	ENSG00000144852	
		MGAM	HBTAR004966	ENSG00000257335	
		ABCG2	HBTAR005220	ENSG00000118777	
		NPEPPS	HBTAR005272	ENSG00000141279	
		NR1I3	HBTAR005548	ENSG00000143257	
		ACAA2	HBTAR005834	ENSG00000167315	
		AHSA1	HBTAR005931	ENSG00000100591	
		RASSF1	HBTAR006247	ENSG00000068028	
		CHEK2	HBTAR006254	ENSG00000183765	
		DUOX2	HBTAR007419	ENSG00000140279	
		HERC5	HBTAR007584	ENSG00000138646	
		NCF1	HBTAR012441	ENSG00000158517	
		PIM1	HBTAR003005	ENSG00000137193	
		F7	HBTAR001307	ENSG00000057593	
		DPEP1	HBTAR001094	ENSG00000015413	
		GABRA1	HBTAR001471	ENSG00000022355	
		CDK1	HBTAR000607	ENSG00000170312	
		DPP4	HBTAR001096	ENSG00000197635	
		CA2	HBTAR000460	ENSG00000104267	
		MAPK14	HBTAR000874	ENSG00000112062	
		CDK2	HBTAR000634	ENSG00000123374	
		CCNA2	HBTAR000549	ENSG00000145386	
		CHEK1	HBTAR000686	ENSG00000149554	
		NCOA2	HBTAR005869	ENSG00000140396	
Cholesterol	HBIN021150	NR3C2	HBTAR002504	ENSG00000151623	8
		PGR	HBTAR002971	ENSG00000082175	
		NCOA2	HBTAR005869	ENSG00000140396	
		DPP4	HBTAR001096	ENSG00000197635	
		ESR1	HBTAR001277	ENSG00000091831	
		AR	HBTAR000233	ENSG00000169083	
		NR3C1	HBTAR001692	ENSG00000113580	
		PRSS1	HBTAR003223	ENSG00000204983	
Beta-carotene	HBIN018006	AKT1	HBTAR000130	ENSG00000142208	21
		ALB	HBTAR000135	ENSG00000163631	
		BCL2	HBTAR000360	ENSG00000171791	
		CASP3	HBTAR000517	ENSG00000164305	
		CASP7	HBTAR000521	ENSG00000165806	

		CASP8	HBTAR000522	ENSG00000064012	
		CASP9	HBTAR000523	ENSG00000132906	
		CAV1	HBTAR000529	ENSG00000105974	
		CTNNB1	HBTAR000920	ENSG00000168036	
		CYP1A2	HBTAR000949	ENSG00000140505	
		CYP3A4	HBTAR000964	ENSG00000160868	
		F3	HBTAR001305	ENSG00000117525	
		GJA1	HBTAR001563	ENSG00000152661	
		HMOX1	HBTAR001851	ENSG00000100292	
		JUN	HBTAR002166	ENSG00000177606	
		MMP1	HBTAR002507	ENSG00000196611	
		MMP2	HBTAR002508	ENSG00000087245	
		MMP10	HBTAR002513	ENSG00000166670	
		MYC	HBTAR002597	ENSG00000136997	
		PTGS2	HBTAR003295	ENSG00000073756	
		VEGFA	HBTAR004310	ENSG00000112715	
Sitosterol	HBIN044518	APOE	HBTAR000218	ENSG00000130203	22
		BAX	HBTAR000351	ENSG00000087088	
		BCL2	HBTAR000360	ENSG00000171791	
		CASP3	HBTAR000517	ENSG00000164305	
		CASP8	HBTAR000522	ENSG00000064012	
		CASP9	HBTAR000523	ENSG00000132906	
		DHCR24	HBTAR001043	ENSG00000116133	
		ICAM1	HBTAR001976	ENSG00000090339	
		JUN	HBTAR002166	ENSG00000177606	
		MAP2	HBTAR002406	ENSG00000078018	
		NR3C2	HBTAR002504	ENSG00000151623	
		PGR	HBTAR002971	ENSG00000082175	
		PON1	HBTAR003098	ENSG00000005421	
		PRKCA	HBTAR003177	ENSG00000154229	
		SREBF1	HBTAR003895	ENSG00000072310	
		SREBF2	HBTAR003896	ENSG00000198911	
		TGFB1	HBTAR004072	ENSG00000105329	
		ABCB11	HBTAR004788	ENSG00000073734	
		NR3C1	HBTAR001692	ENSG00000113580	
		ESR1	HBTAR001277	ENSG00000091831	
		NCOA2	HBTAR005869	ENSG00000140396	
		AR	HBTAR000233	ENSG00000169083	
Solasonine	HBIN044320	ATP1A1	HBTAR000292	ENSG00000163399	1
Solamargine	HBIN044265	BCL2	HBTAR000360	ENSG00000171791	11
		BCL2L1	HBTAR000362	ENSG00000171552	
		CASP3	HBTAR000517	ENSG00000164305	
		ERBB2	HBTAR001263	ENSG00000141736	
		LTA	HBTAR002356	ENSG00000226979	
		TNF	HBTAR004140	ENSG00000232810	

PSG1	HBRAR003237	ENSG00000231924
PTGER4	HBTAR003288	ENSG00000171522
RELA	HBTAR003434	ENSG00000173039
NFKB1	HBTAR002705	ENSG00000109320
AKT1	HBTAR000130	ENSG00000142208

Individual target genes corresponding to the selected active compounds of SN were obtained from the HERB database (http://herb.ac.cn/).

Table S4. Top 10 enriched Gene Ontology (GO) terms categorized by biological process (BP), cellular component (CC), and molecular function (MF).

Term	Count	PValue	Genes	Fold Enrichment	Class
positive regulation of apoptotic process	10	3.03.E-11	PTGS2/BCL2/CASP8/MMP9/TGFB1/TNF/BAX/IL6/JUN/CASP9	26.65	GO BP
positive regulation of gene expression	10	1.74.E-09	VEGFA/GJA1/CAV1/HIF1A/TGFB1/TNF/ERBB2/IL6/F3/RELA	16.86	
response to xenobiotic stimulus	9	1.85.E-10	CCND1/PTGS2/BCL2/TGFB1/TNF/CASP3/MMP2/IL10/RELA	30.40	
response to hypoxia	8	6.31.E-10	VEGFA/CAV1/HIF1A/TGFB1/TNF/CASP3/MMP2/CASP9	38.78	
negative regulation of apoptotic process	8	8.65.E-07	VEGFA/BCL2/MMP9/TNF/ERBB2/IL6/IL10/RELA	13.54	
positive regulation of canonical NF-kappaB signal transduction	7	1.39.E-07	VEGFA/GJA1/CAV1/CASP8/TGFB1/TNF/RELA	26.46	
angiogenesis	7	2.88.E-07	VEGFA/PTGS2/CAV1/HIF1A/CASP8/MMP2/PRKCA	23.37	
positive regulation of cell migration	7	5.51.E-07	VEGFA/CAV1/CASP8/TGFB1/MMP2/PRKCA/F3	20.93	
response to glucocorticoid	6	2.17.E-09	PTGS2/BCL2/TNF/CASP3/IL6/IL10	102.96	
positive regulation of vascular associated smooth muscle cell proliferation	6	2.93.E-09	GJA1/MMP9/TNF/MMP2/JUN/IL10	97.13	
cytoplasm	14	1.87.E-03	VEGFA/PTGS2/GJA1/CASP8/BCL2/TGFB1/PRKCA/RELA/CCND1/HIF1A/CASP3/ERBB2/BAX/CASP9	2.21	GO CC
nucleus	14	3.74.E-03	VEGFA/GJA1/BCL2/TGFB1/PRKCA/JUN/RELA/CCND1/HIF1A/CASP3/ERBB2/BAX/MMP2/CASP9	2.06	
cytosol	11	4.89.E-02	CCND1/GJA1/HIF1A/BCL2/CASP8/ERBB2/CASP3/BAX/PRKCA/CASP9/RELA	1.77	
extracellular space	9	7.28.E-04	VEGFA/MMP9/TGFB1/TNF/IL6/MMP2/F3/MMP1/IL10	4.02	
nucleoplasm	9	4.65.E-02	CCND1/GJA1/HIF1A/CASP8/ERBB2/CASP3/PRKCA/JUN/RELA	2.03	
extracellular region	8	4.51.E-03	VEGFA/MMP9/TGFB1/TNF/IL6/MMP2/MMP1/IL10	3.46	
endoplasmic reticulum	7	1.12.E-03	VEGFA/PTGS2/GJA1/CAV1/BCL2/BAX/PRKCA	5.36	
mitochondrion	7	3.54.E-03	GJA1/BCL2/CASP8/BAX/MMP2/PRKCA/CASP9	4.28	
protein-containing complex	6	6.15.E-04	PTGS2/CAV1/HIF1A/BCL2/CASP8/CASP9	7.97	
extracellular matrix	5	1.06.E-04	VEGFA/MMP9/TGFB1/MMP2/MMP1	18.92	
protein binding	22	1.34.E-03	VEGFA/PTGS2/GJA1/CAV1/CASP8/BCL2/TGFB1/PRKCA/JUN/IL6/F3/IL10/RELA/CCND1/HIF1A/MMP9/TNF/CASP3/ERBB2/BAX/MMP2/CASP9	1.45	GO MF
identical protein binding	12	8.40.E-07	VEGFA/CAV1/BCL2/CASP8/MMP9/TGFB1/TNF/ERBB2/BAX/JUN/CASP9/RELA	5.80	
enzyme binding	8	1.24.E-07	CCND1/PTGS2/CAV1/HIF1A/TGFB1/PRKCA/JUN/RELA	18.05	
peptidase activity	5	4.26.E-06	CASP8/MMP9/CASP3/MMP1/CASP9	42.82	

cytokine activity	5	6.40.E-05	VEGFA/TGFB1/TNF/IL6/IL10	21.52
ubiquitin protein ligase binding	5	3.84.E-04	HIF1A/BCL2/CASP8/JUN/RELA	13.49
protein heterodimerization activity	5	8.00.E-04	CAV1/HIF1A/BCL2/ERBB2/BAX	11.10
protein kinase binding	5	2.28.E-03	CCND1/CAV1/HIF1A/CASP9/RELA	8.36
protein homodimerization activity	5	9.46.E-03	VEGFA/PTGS2/BCL2/BAX/RELA	5.60
protease binding	4	3.15.E-04	BCL2/TNF/CASP3/F3	28.45

GO enrichment analysis was performed using the DAVID database (https://david.ncifcrf.gov/). The enriched terms were selected based on a significance threshold of $p < 0.05$ and subsequently ranked by gene count to identify the top 10 terms per category.

Table S5. Top 20 enriched KEGG pathways associated with the predicted targets.

Term	Count	PValue	Genes	Fold Enrichment
Pathways in cancer	18	2.81.E-17	VEGFA/PTGS2/CASP8/BCL2/TGFB1/PRKCA/JUN/IL6/MMP1/ RELA/CCND1/HIF1A/MMP9/CASP3/ERBB2/BAX/MMP2/CASP9	12.98
AGE-RAGE signaling pathway in diabetic complications	13	1.48.E-18	VEGFA/BCL2/TGFB1/PRKCA/JUN/IL6/F3/RELA/CCND1/TNF/ CASP3/BAX/MMP2	49.47
Hepatitis B	12	3.53.E-14	BCL2/CASP8/MMP9/TGFB1/TNF/CASP3/BAX/IL6/PRKCA/JUN/ CASP9/RELA	28.30
Lipid and atherosclerosis	12	8.02.E-13	BCL2/CASP8/MMP9/TNF/CASP3/BAX/IL6/PRKCA/JUN/MMP1/ CASP9/RELA	21.35
Kaposi sarcoma-associated herpesvirus infection	11	1.17.E-11	CCND1/VEGFA/PTGS2/HIF1A/CASP8/CASP3/BAX/IL6/JUN/ CASP9/RELA	21.57
Proteoglycans in cancer	11	1.75.E-11	CCND1/VEGFA/CAV1/HIF1A/MMP9/TGFB1/TNF/ERBB2/CASP3/MMP2/PRKCA	20.72
Human cytomegalovirus infection	11	4.84.E-11	CCND1/VEGFA/PTGS2/CASP8/TNF/CASP3/BAX/IL6/PRKCA/ CASP9/RELA	18.71
Tuberculosis	10	1.95.E-10	BCL2/CASP8/TGFB1/TNF/CASP3/BAX/IL6/CASP9/IL10/RELA	21.35
Epstein-Barr virus infection	10	5.72.E-10	CCND1/BCL2/CASP8/TNF/CASP3/BAX/IL6/JUN/CASP9/RELA	18.93
IL-17 signaling pathway	9	3.73.E-11	PTGS2/CASP8/MMP9/TNF/CASP3/IL6/JUN/MMP1/RELA	36.41
Measles	9	8.11.E-10	CCND1/BCL2/CASP8/CASP3/BAX/IL6/JUN/CASP9/RELA	24.89
Human immunodeficiency virus 1 infection	9	2.38.E-08	BCL2/CASP8/TNF/CASP3/BAX/PRKCA/JUN/CASP9/RELA	16.24
Toxoplasmosis	8	5.94.E-09	BCL2/CASP8/TGFB1/TNF/CASP3/CASP9/IL10/RELA	27.70
TNF signaling pathway	8	9.68.E-09	PTGS2/CASP8/MMP9/TNF/CASP3/IL6/JUN/RELA	25.84
Relaxin signaling pathway	8	1.80.E-08	VEGFA/MMP9/TGFB1/MMP2/PRKCA/JUN/MMP1/RELA	23.65
Apoptosis	8	2.46.E-08	BCL2/CASP8/TNF/CASP3/BAX/JUN/CASP9/RELA	22.61
Fluid shear stress and atherosclerosis	8	3.16.E-08	VEGFA/CAV1/BCL2/MMP9/TNF/MMP2/JUN/RELA	21.81
Pancreatic cancer	7	2.39.E-08	CCND1/VEGFA/TGFB1/ERBB2/BAX/CASP9/RELA	34.94
Colorectal cancer	7	5.02.E-08	CCND1/BCL2/TGFB1/CASP3/BAX/JUN/CASP9	30.92
Bladder cancer	6	4.15.E-08	CCND1/VEGFA/MMP9/ERBB2/MMP2/MMP1	56.25

KEGG pathway enrichment analysis was conducted using the DAVID database (https://david.ncifcrf.gov/). The top 20 pathways were filtered using a significance threshold of $p < 0.05$, organized by gene count.

Table S6. Comprehensive list of the 23 overlapping target genes mapped between OVCA, SN, and its 6 key active compounds.

Compounds	Ingredient ID	Target name	Target ID	Ensembl ID
Diosgenin	HBIN024164	HIF1A	HBTAR001810	ENSG00000100644
		PTGS2	HBTAR003295	ENSG00000073756
		RELA	HBTAR003434	ENSG00000173039
		VEGFA	HBTAR004310	ENSG00000112715
Sitosterol	HBIN044518	BAX	HBTAR000351	ENSG00000087088
		BCL2	HBTAR000360	ENSG00000171791
		CASP3	HBTAR000517	ENSG00000164305
		CASP8	HBTAR000522	ENSG00000064012
		CASP9	HBTAR000523	ENSG00000132906
		JUN	HBTAR002166	ENSG00000177606
		PRKCA	HBTAR003177	ENSG00000154229
		TGFB1	HBTAR004072	ENSG00000105329
Medioresinol	HBIN009785	PTGS2	HBTAR003295	ENSG00000073756
Beta-carotene	HBIN018006	BCL2	HBTAR000360	ENSG00000171791
		CASP3	HBTAR000517	ENSG00000164305
		CASP8	HBTAR000522	ENSG00000064012
		CASP9	HBTAR000523	ENSG00000132906
		CAV1	HBTAR000529	ENSG00000105974
		F3	HBTAR001305	ENSG00000117525
		GJA1	HBTAR001563	ENSG00000152661
		JUN	HBTAR002166	ENSG00000177606
		MMP1	HBTAR002507	ENSG00000196611
		MMP2	HBTAR002508	ENSG00000087245
		PTGS2	HBTAR003295	ENSG00000073756
		VEGFA	HBTAR004310	ENSG00000112715
Solamargine	HBIN044265	BCL2	HBTAR000360	ENSG00000171791
		CASP3	HBTAR000517	ENSG00000164305
		ERBB2	HBTAR001263	ENSG00000141736
		TNF	HBTAR004140	ENSG00000232810
		RELA	HBTAR003434	ENSG00000173039
Quercetin	HBIN041495	BAX	HBTAR000351	ENSG00000087088
		CCND1	HBTAR000359	ENSG00000110092
		BCL2	HBTAR000360	ENSG00000171791
		CASP3	HBTAR000517	ENSG00000164305
		CASP8	HBTAR000522	ENSG00000064012
		CASP9	HBTAR000523	ENSG00000132906
		CAV1	HBTAR000529	ENSG00000105974
		ERBB2	HBTAR001263	ENSG00000141736
		F3	HBTAR001305	ENSG00000117525
		GJA1	HBTAR001563	ENSG00000152661
		HIF1A	HBTAR001810	ENSG00000100644

IL6	HBTAR002055	ENSG00000136244
IL10	HBTAR002064	ENSG00000136634
JUN	HBTAR002166	ENSG00000177606
MMP1	HBTAR002507	ENSG00000196611
MMP2	HBTAR002508	ENSG00000087245
MMP9	HBTAR002512	ENSG00000100985
PRKCA	HBTAR003177	ENSG00000154229
PTGS2	HBTAR003295	ENSG00000073756
RELA	HBTAR003434	ENSG00000173039
TGFB1	HBTAR004072	ENSG00000105329
TNF	HBTAR004140	ENSG00000232810
VEGFA	HBTAR004310	ENSG00000112715

The intersection of target genes was identified and mapped using the Venny (v2.1.0) software. Detailed target gene annotations for each overlapping compound were retrieved from the HERB database (http://herb.ac.cn/).

Table S7. Topological centrality analysis of core target genes involved in the "Pathways in cancer" network.

Target name	Degree centrality	Betweenness centrality	Closeness centrality
CASP3	12	63.9992	0.0455
IL6	12	25.0317	0.0455
JUN	12	25.3333	0.0455
VEGFA	11	11.4087	0.0435
MMP9	11	11.4087	0.0435
HIF1A	9	6.3460	0.0400
CCND1	9	13.6317	0.0400
RELA	7	12.7651	0.0370
MMP2	7	1.8833	0.0333
TGFB1	7	2.7603	0.0345

Protein-protein interaction (PPI) data were generated using the STRING database (https://string-db.org/) with the organism set to Homo sapiens and a high-confidence interaction score threshold of > 0.700. Topological parameters, such as Degree Centrality (DC), Betweenness Centrality (BC), and Closeness Centrality (CC), were calculated using the CentiScaPe plugin (v2.2).

Table S8. HPLC analysis of solasonine in SN extract and its fractions.

Batch	Solasonine[1]		
	**RT (min)** [2]	**mg/g**[2]	**SD**[2]
Crude extract	4.702	0.108	0.002
n-hexane fr.	4.714	0.740	0.005
CH_2Cl_2 fr.	4.745	0.045	0.001
EtOAc fr.	4.723	0.248	0.001
n-BuOH fr.	4.861	0.216	0.003
aqueous layer	4.756	-	-

[1] Solasonine, a structural analog of the *in silico* target solamargine, was used as a quantitative marker. [2] Retention times (RT), contents (mg/g), and standard deviations (SD) determined by HPLC analysis. "–" indicates that the compound was not detected.
